# Supplementary material for: Nonlinear Fourier classification of 663 rogue waves measured in the Philippine Sea
Source: PLoS One. 2024 May 14;19(5):e0301709. doi: 10.1371/journal.pone.0301709 (PMC11093313; doi:10.1371/journal.pone.0301709)
Supplement: S1 File — This file contains additional information for identifying rogue waves from the solutions of the NLSE-NFT. (PDF) [file pone.0301709.s001.pdf]

Supporting Information S1

for "Nonlinear Fourier Classification of 663 Rogue Waves Measured in the Philippine Sea"

Y.-C. Lee, M. Brühl, D.J. Doong and S. Wahls

1 Localization of Nonlinear Modes for Periodic Nonlinear Fourier Analysis of Rogue Waves

Regarding the question of identification of rogue waves from the solutions of NLSE, the interpretation of spectral portraits of nonlinear spectra is a ongoing topic of discussion. Several studies provide the nonlinear spectra of rogue waves that have been removed from their surroundings [1–3]. These localized structures are selected from the wave trains of interest, and directly used for the input of NLSE-NFT. The reduced sampling frequency or number of points may however influence the frequency resolution. Here, we propose a new signal pre-processing approach for localized structures from wave trains when applying periodic NFT.

Our approach ensures that the nonlinear frequency resolution stays fixed, which is similar to zero-padding technique in conventional FFT. Using the given time series with a rogue wave, the approach consists of the following steps: (1) Find the maximum wave height in the wave train. (2) Define the desired width of truncated section and keep the rogue wave in the center if possible. (3) Set the time series to zero outside the truncated section so that the original length is preserved. (4) Compute the nonlinear spectrum of the resulting time series.

2 Results of the Localization Procedure

In this document, we provide in total 24 examples with four types of nonlinear spectra in order to provide more evidence of our finding that the largest modes in soliton- and large breather-type spectra are associated to the rogue wave because they are retained under the localization procedure described above, and that this is not the case for stable- and small breather-type spectra.

We now apply the localization procedure to four exemplary rogue wave samples to illustrate the findings (one per type). The remaining 20 examples are shown at the end for further verification.

2.1 Stable Mode Example

In this stable mode example, the rogue wave sample was measured from 10:00h on 02 November 2009 with duration of 600 s. The maximum amplitude of rogue wave occurs at 327 s in the time series. The truncation window sizes are chosen as as 75 s, 150 s, 300 s, 450s. Following the above approach, we show all time series in Figure 1. The corresponding nonlinear spectra (obtained by NLSE-NFT) are shown in Figure 2. All spectra belong to type 1: stable mode. This indicates that the stable modes in nonlinear spectra compose the rogue wave in the time series.

2.2 Small Breather Example

The time series and nonlinear spectra are presented in Figure 3 and Figure 4, respectively. The original time series was measured at 06:00h on 17 November 2011. The maximum amplitude of the rogue wave occurs at 210 s. The nonlinear spectra in Figure 4a and 4b of the time series with shorter truncated temporal structures consist of stable modes. There is one small breather observed in Figure 4c with coordinates (0.082 Hz, 0.28 m), (0.083 Hz, 0.30 m). When the length of truncated structure increases to 450 s, the spectrum changes and two breathers appear with coordinates (0.089 Hz, 0.36 m), (0.090 Hz, 0.30 m) and (0.097 Hz, 0.39 m), (0.098 Hz, 0.38 m) as shown in Figure 4d. However, the spectrum of entire time series in Figure 4e only remains one breather with coordinates (0.089 Hz, 0.36 m), (0.090 Hz, 0.30 m) and the other breather degenerates into two large spines. The nonlinear spectrum still seems to imply that the small breather is relevant to the rogue wave in the time series, but it cannot be localized close to it. One possible explanation might be that the small breather is wide and thus requires a larger localized frame to be captured.

2.3 Large Breather Example

The time series and nonlinear spectra are presented in Figure 5 and Figure 6, respectively. The original time series was measured at 23:00h on 07 August 2015. The maximum amplitude of the rogue wave occurs at 373 s. The nonlinear spectrum in Figure 6a for the truncated temporal structures of 75 s consists completely of stable modes. When the length of truncated structure increases to 150 s, one breather appears with coordinates (0.067 Hz, 1.12 m), (0.066 Hz, 0.88 m) in Figure 6b. This spectrum is classified as type 3: large breather. With the increasing length of truncated structure, the breather increases the amplitude to around 1.3 m and another small breather appears in Figure 6c-6e. Since the same large breather is visible for all but the strongest truncation, we assume that the short window does not cover the whole nonlinear structure that is attributed to the large breather. This requires more further investigation.

2.4 Soliton Example

The time series and nonlinear spectra are presented in Figure 7 and Figure 8, respectively. The original time series was measured at 05:00h on 27 September 2015. The maximum amplitude of the rogue wave occurs at 69 s. All nonlinear spectra in Figure 8 are classified as type 4: soliton. With the increasing length of the truncated structure from Figure 8a to 8b, the amplitude of the soliton increases from 0.90 m to 1.37 m. Some small breathers appear, but the amplitude of soliton stays similar in Figure 8c to 8e. In Figure 8e, the soliton is located at coordinates (0.091 Hz, 1.32 m). The results indicates that the soliton is directly related to the rogue wave in the time series.

2.5 More Examples

The other 20 examples are shown at the end for more verification. The nonlinear spectra in Figure 9 to Figure 18 belong to type 1: stable mode. Figure 19 to Figure 28 belong to type 2: small breather. Figure 29 to Figure 38 belong to type 3: large breather. Figure 39 to Figure 48 belong to type 4: soliton.

References

[1] Toenger, S. *et al.* Emergent rogue wave structures and statistics in spontaneous modulation instability. *Scientific reports* **5**, 1–8 (2015).

[2] Randoux, S., Suret, P. & El, G. Inverse scattering transform analysis of rogue waves using local periodization procedure. *Scientific reports* **6**, 1–11 (2016).

[3] Onorato, M. *et al.* Observation of a giant nonlinear wave-packet on the surface of the ocean. *Scientific Reports* **11**, 1–7 (2021).

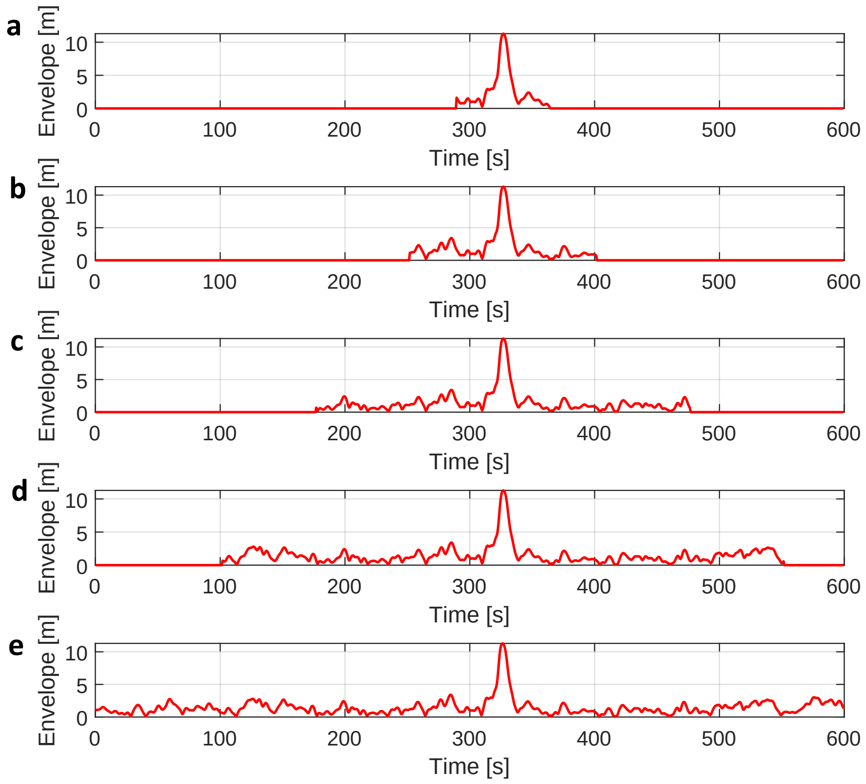

Figure 1: Stable-mode type example: Time series of magnitude of complex envelope of rogue wave records from Taitung Open Ocean buoy measured at 10:00h on 02 November 2009. The maximum amplitude of rogue wave occurs at 327 s in the time series. The original signal of 600 s (time series **e**) is cut into different time length 75 s, 150 s, 300 s, 450 s, and sets the zero outside windows of duration as shown in **a** to **d**, respectively.

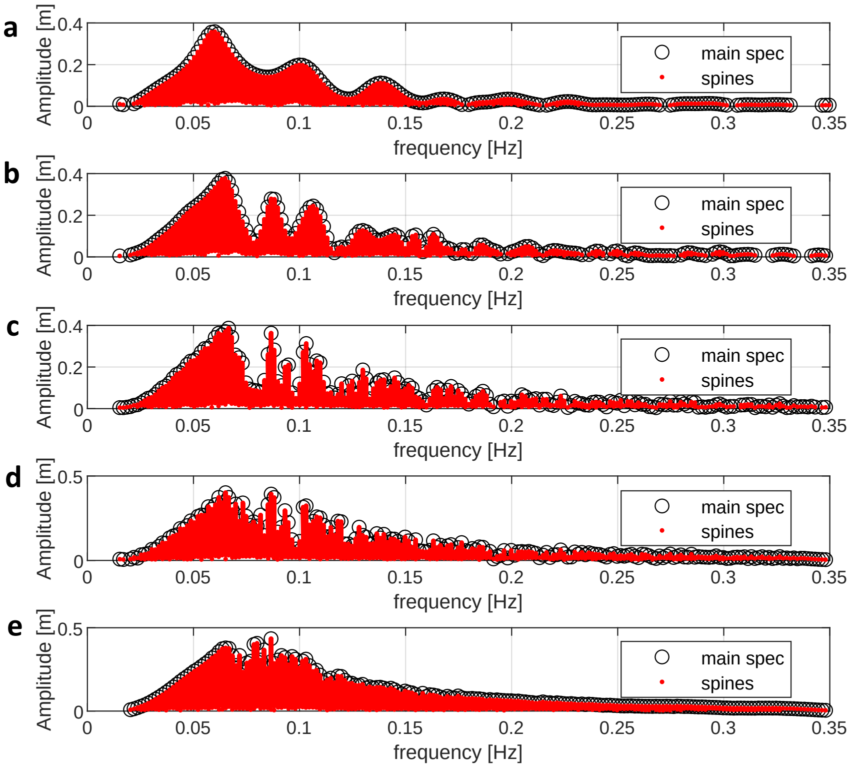

Figure 2: Stable-mode type example: Nonlinear spectra from **a** to **e** are obtained by NLSE-NFT with the magnitude of complex envelope in Figure 1 from **a** to **e**. The nonlinear spectrum of the time series in Figure 1e is classified as type 1: stable-mode spectrum.

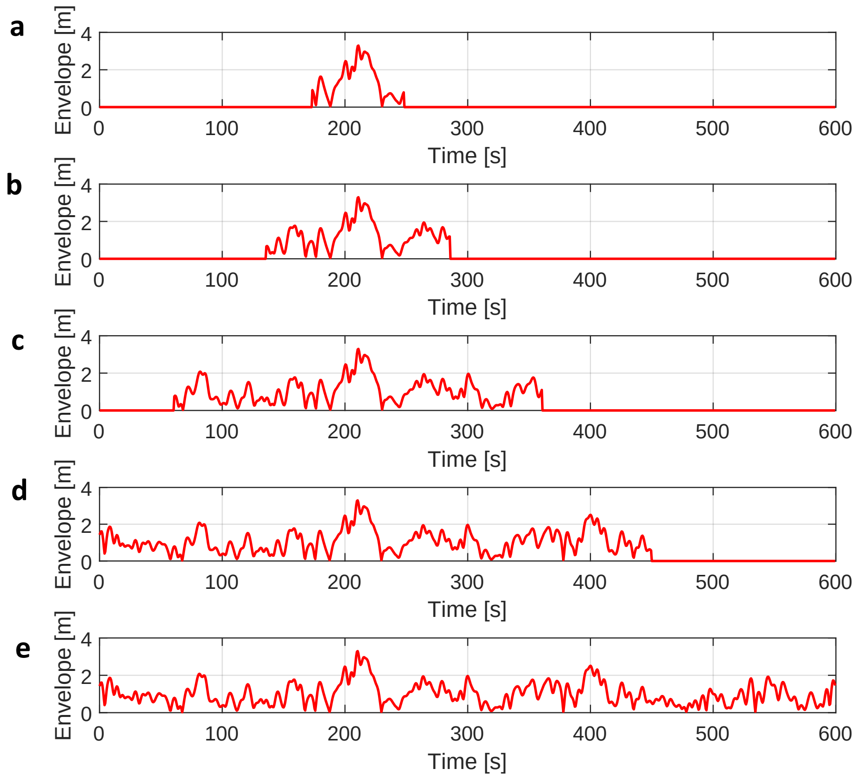

Figure 3: Small breather type example: Time series of magnitude of complex envelope of rogue wave records from Taitung Open Ocean buoy measured from 06:00h on 17 November 2011. The maximum amplitude of rogue wave occurs at 210 s in the time series. The original signal of 600 s (time series **e**) is cut into different time length 75 s, 150 s, 300 s, 450 s, and sets the zero outside windows of duration as shown in **a** to **d**, respectively.

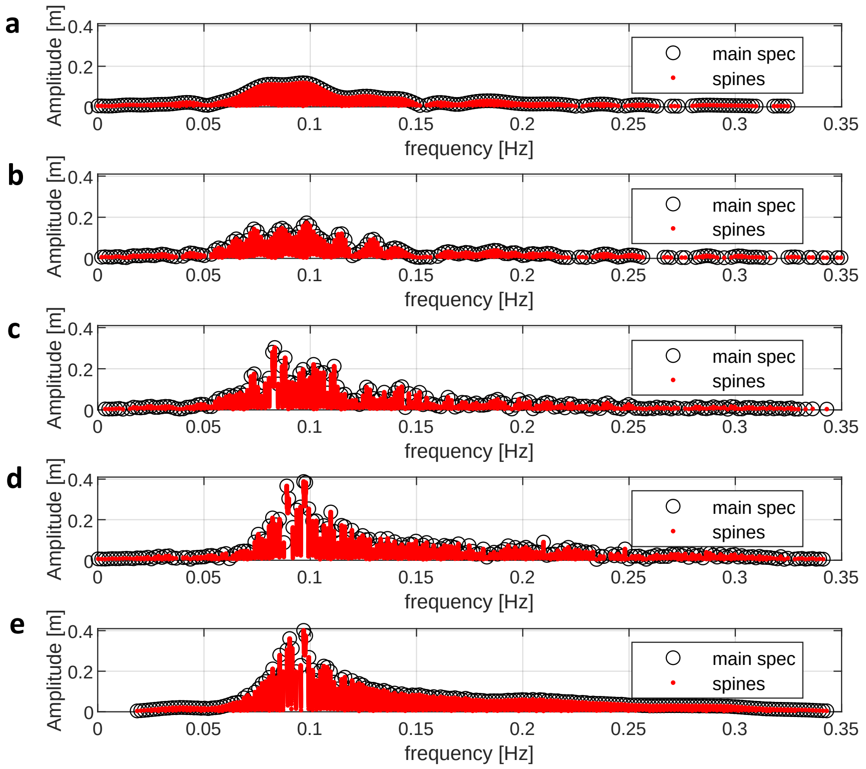

Figure 4: Small breather type example: Nonlinear spectra from **a** to **e** are obtained by NLSE-NFT with the magnitude of complex envelope in Figure 3 from **a** to **e**. The nonlinear spectrum of the time series in Figure 3e is classified as type 2: small breather spectrum.

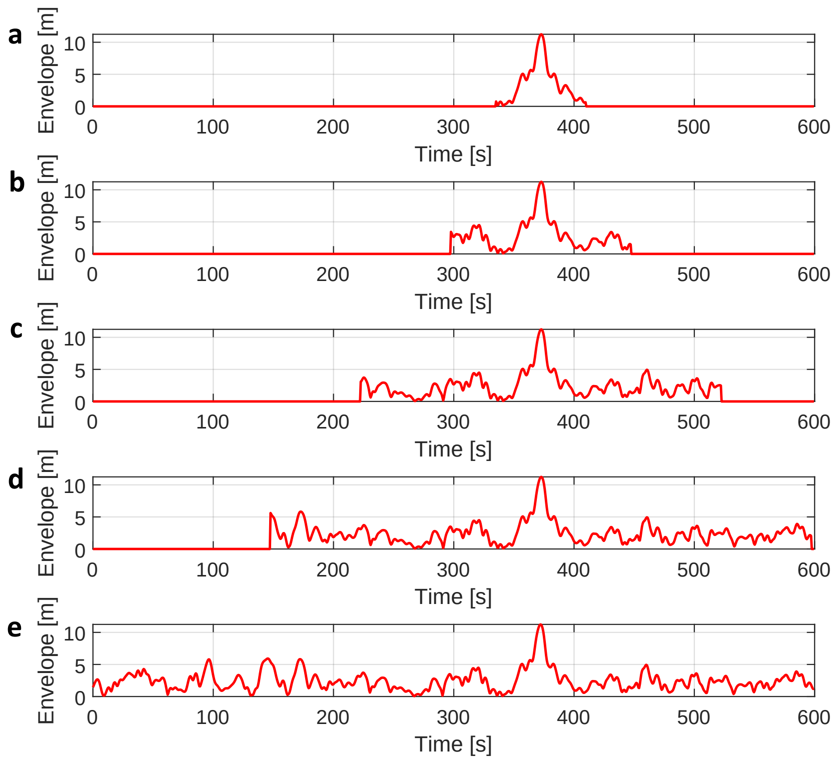

Figure 5: Large breather type example: Time series of magnitude of complex envelope of rogue wave records from Taitung Open Ocean buoy measured from 23:00h on 07 August 2015. The maximum amplitude of rogue wave occurs at 373 s in the time series. The original signal of 600 s (time series **e**) is cut into different time length 75 s, 150 s, 300 s, 450 s, and sets the zero outside windows of duration as shown in **a** to **d**, respectively.

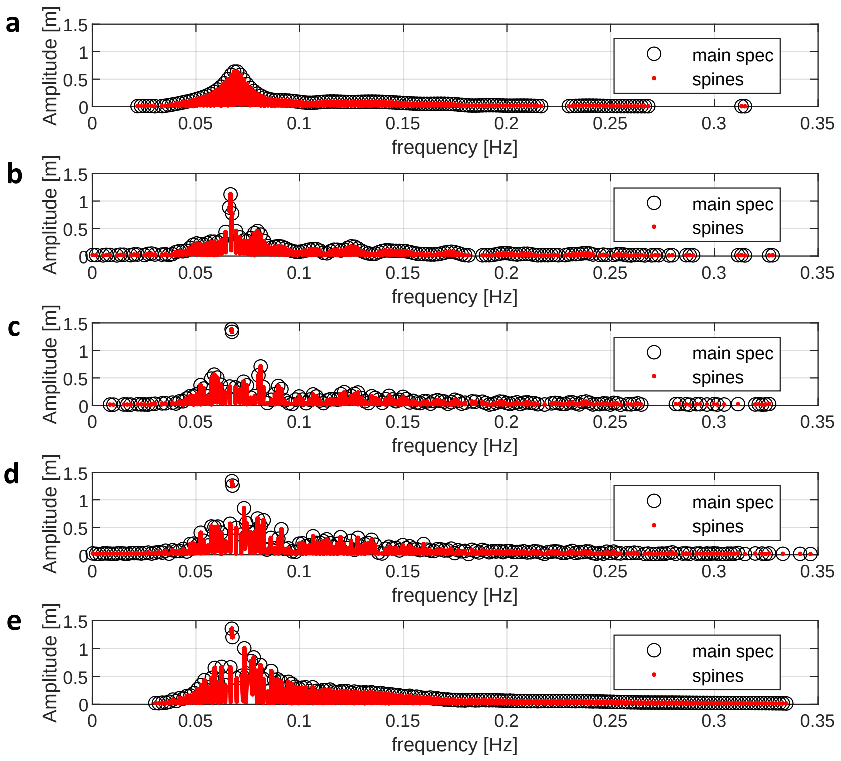

Figure 6: Large breather type example: Nonlinear spectra from **a** to **e** are obtained by NLSE-NFT with the magnitude of complex envelope in Figure 5 from **a** to **e**. The nonlinear spectrum of the time series in Figure 5e is classified as type 3: large breather spectrum.

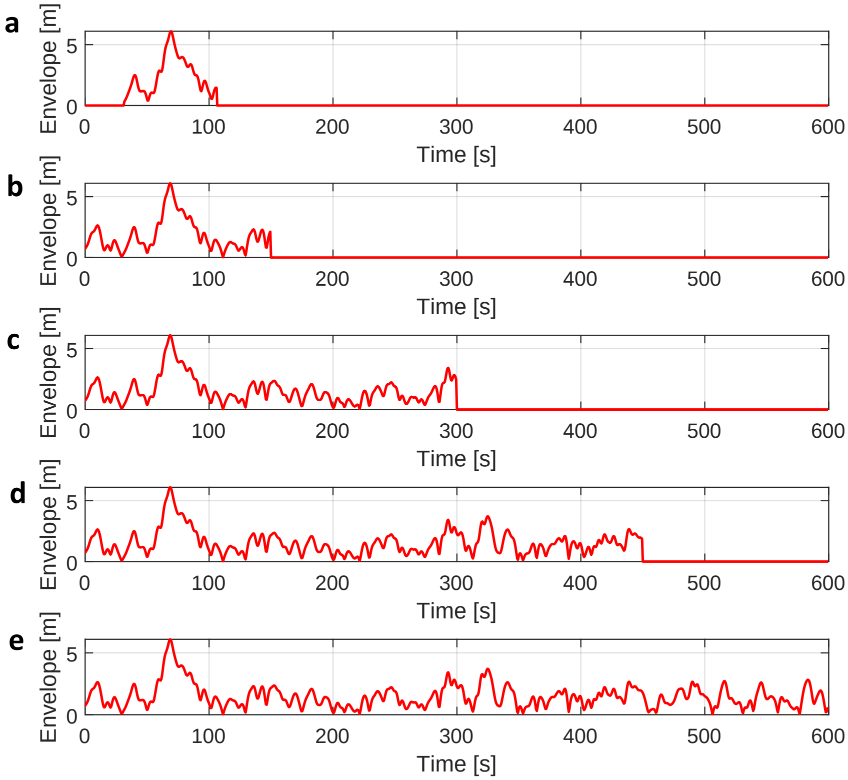

Figure 7: Soliton type example: Time series of magnitude of complex envelope of rogue wave records from Taitung Open Ocean buoy measured from 05:00h on 27 September 2015. The maximum amplitude of rogue wave occurs at 69 s in the time series. The original signal of 600 s (time series **e**) is cut into different time length 75 s, 150 s, 300 s, 450 s, and sets the zero outside windows of duration as shown in **a** to **d**, respectively.

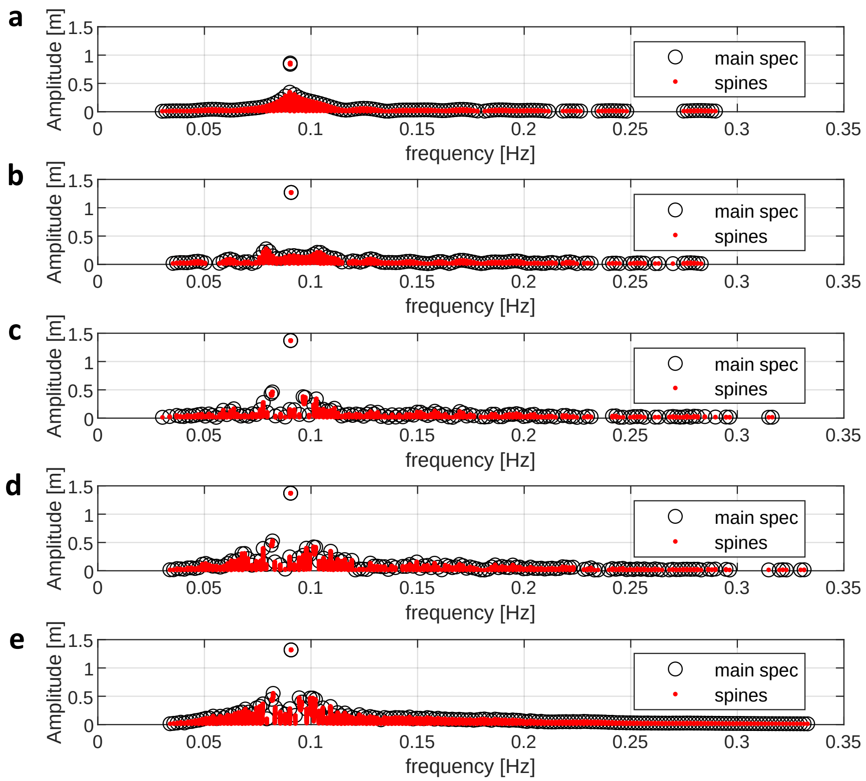

Figure 8: Soliton type example: Nonlinear spectra from **a** to **e** are obtained by NLSE-NFT with the magnitude of complex envelope in Figure 7 from **a** to **e**. The nonlinear spectrum of the time series in Figure 7e is classified as type 4: soliton spectrum.

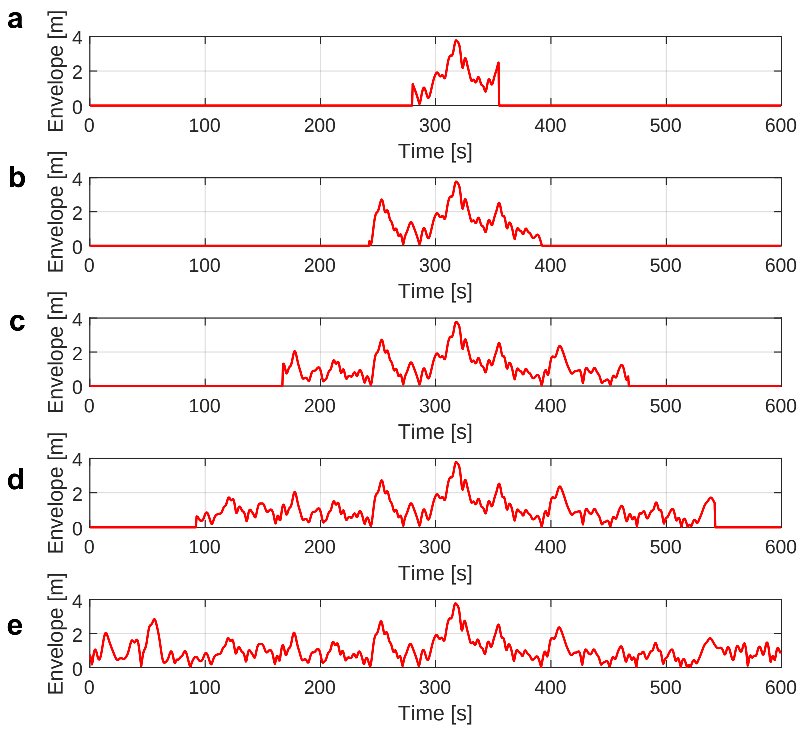

Figure 9: Additional example No. 1 for a stable-mode sample: Time series of magnitude of complex envelope of rogue wave records from Taitung Open Ocean buoy measured from 23:00h on 03 September 2012. The original signal of 600 s (time series **e**) is cut into different time length 75 s, 150 s, 300 s, 450 s, and sets the zero outside windows of duration as shown in **a** to **d**, respectively.

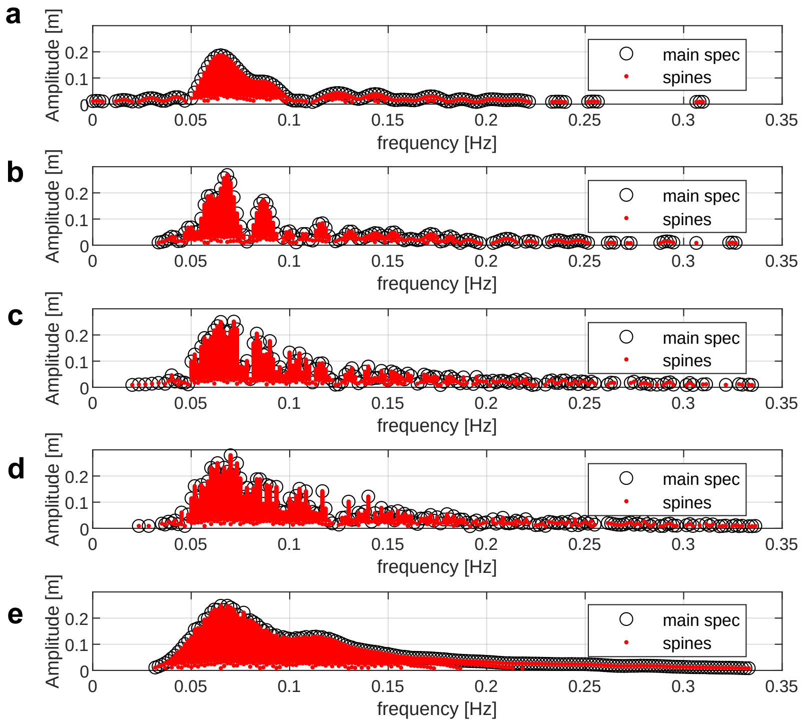

Figure 10: Additional example No. 1 for a stable-mode sample: Nonlinear spectra from **a** to **e** are obtained by NLSE-NFT with the magnitude of complex envelope in Figure 9 from **a** to **e**. The nonlinear spectrum of the time series in Figure 9e is classified as type 1: stable mode spectrum.

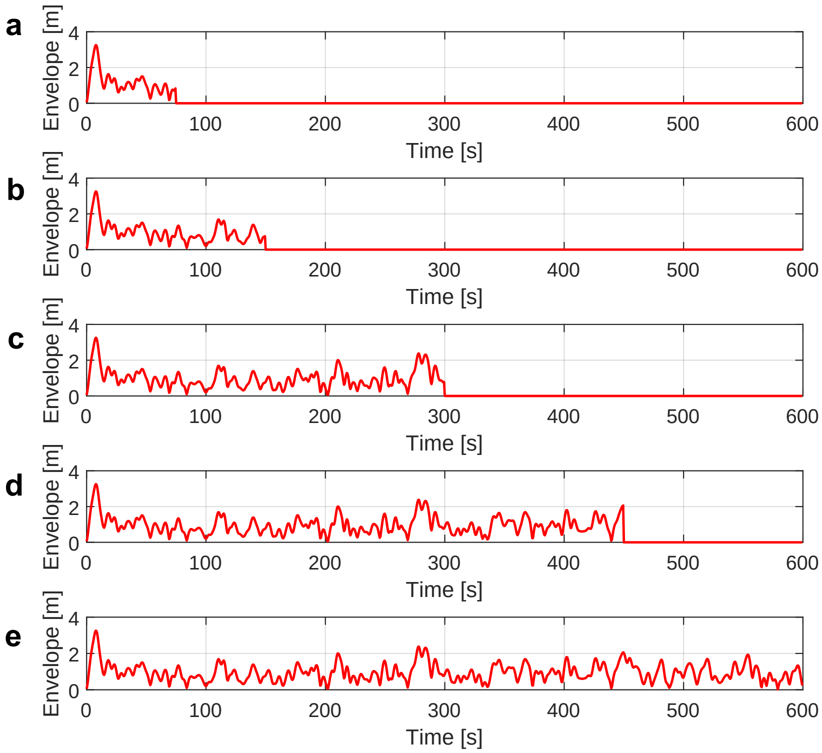

Figure 11: Additional example No. 2 for a stable-mode sample: Time series of magnitude of complex envelope of rogue wave records from Taitung Open Ocean buoy measured from 04:00h on 22 September 2014. The original signal of 600 s (time series **e**) is cut into different time length 75 s, 150 s, 300 s, 450 s, and sets the zero outside windows of duration as shown in **a** to **d**, respectively.

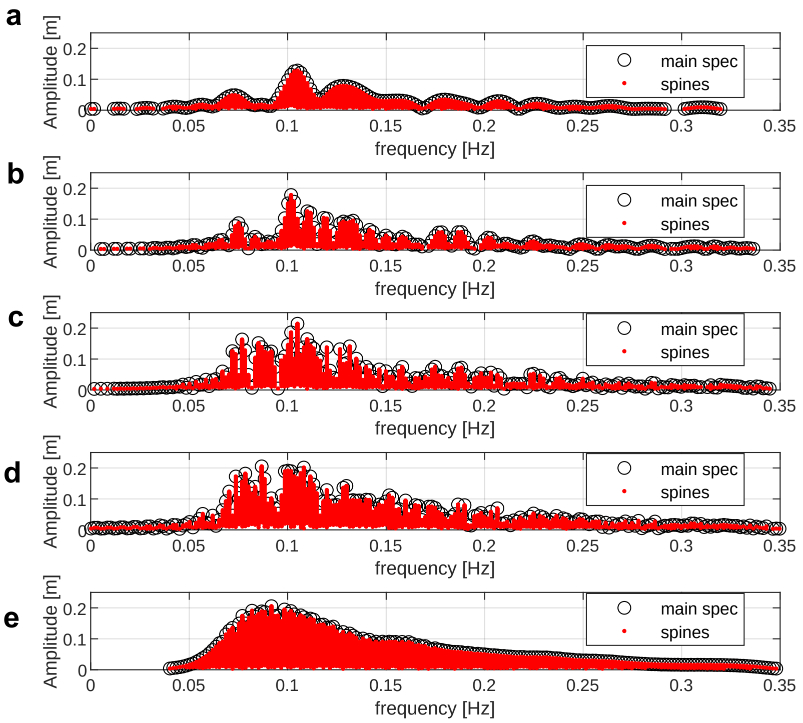

Figure 12: Additional example No. 2 for a stable-mode sample: Nonlinear spectra from **a** to **e** are obtained by NLSE-NFT with the magnitude of complex envelope in Figure 11 from **a** to **e**. The nonlinear spectrum of the time series in Figure 11e is classified as type 1: stable mode spectrum.

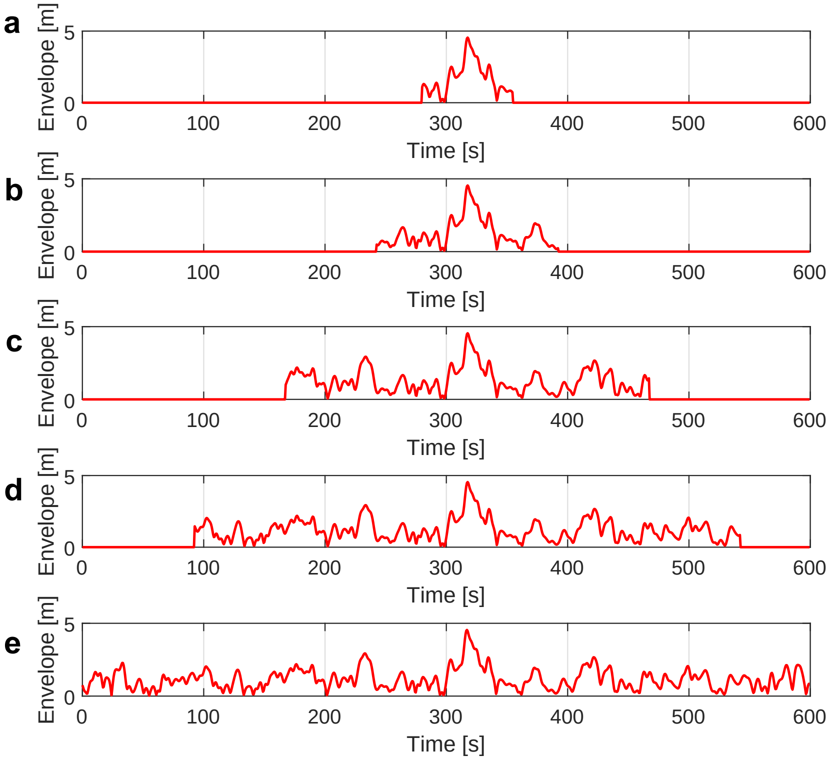

Figure 13: Additional example No. 3 for a stable-mode sample: Time series of magnitude of complex envelope of rogue wave records from Taitung Open Ocean buoy measured from 18:00h on 04 October 2014. The original signal of 600 s (time series **e**) is cut into different time length 75 s, 150 s, 300 s, 450 s, and sets the zero outside windows of duration as shown in **a** to **d**, respectively.

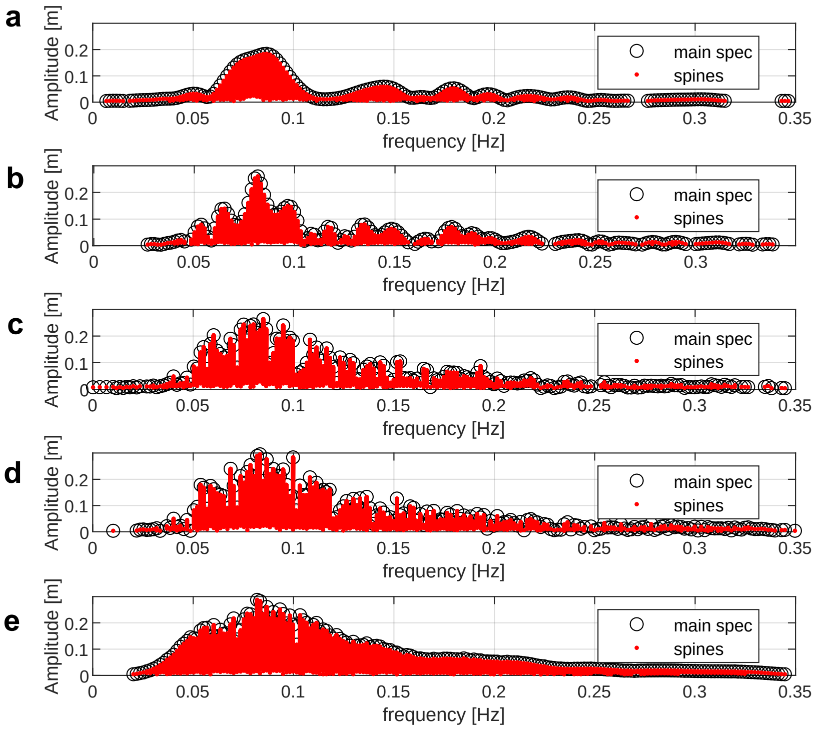

Figure 14: Additional example No. 3 for a stable-mode sample: Nonlinear spectra from **a** to **e** are obtained by NLSE-NFT with the magnitude of complex envelope in Figure 13 from **a** to **e**. The nonlinear spectrum of the time series in Figure 13e is classified as type 1: stable mode spectrum.

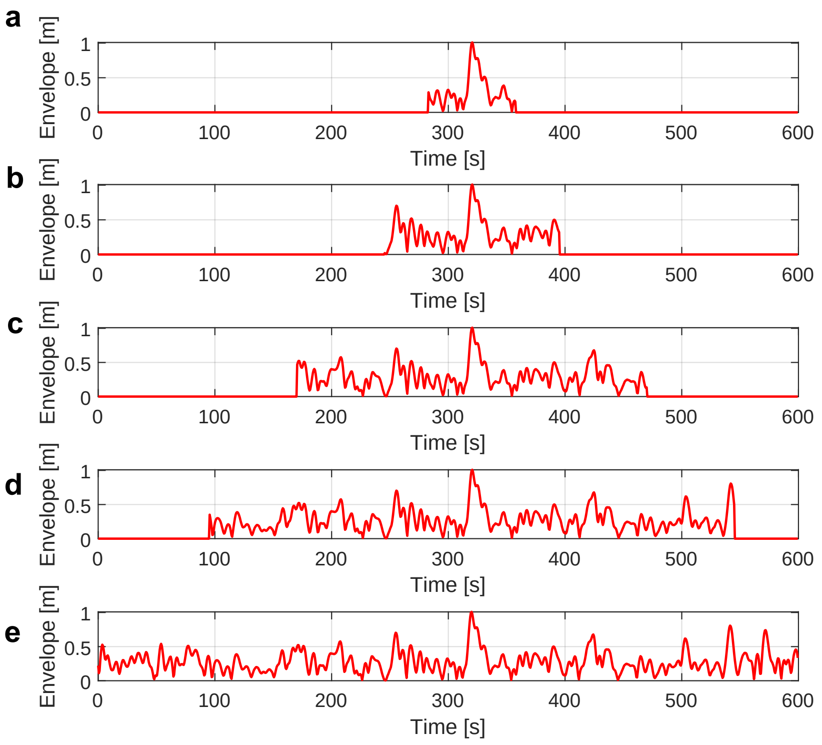

Figure 15: Additional example No. 4 for a stable-mode sample: Time series of magnitude of complex envelope of rogue wave records from Taitung Open Ocean buoy measured from 15:00h on 14 June 2015. The original signal of 600 s (time series **e**) is cut into different time length 75 s, 150 s, 300 s, 450 s, and sets the zero outside windows of duration as shown in **a** to **d**, respectively.

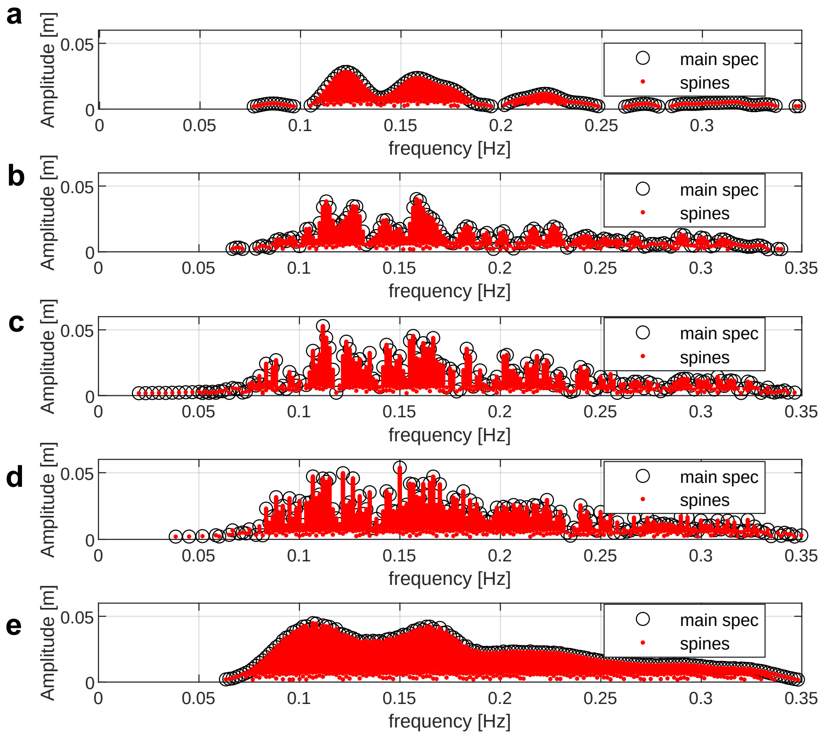

Figure 16: Additional example No. 4 for a stable-mode sample: Nonlinear spectra from **a** to **e** are obtained by NLSE-NFT with the magnitude of complex envelope in Figure 15 from **a** to **e**. The nonlinear spectrum of the time series in Figure 15e is classified as type 1: stable mode spectrum.

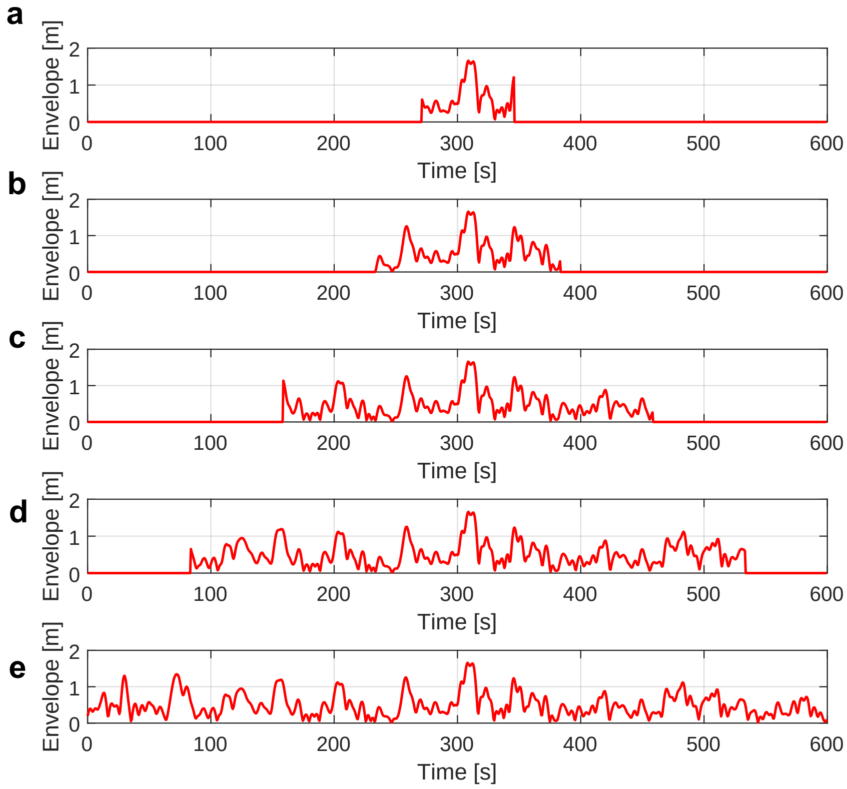

Figure 17: Additional example No. 5 for a stable-mode sample: Time series of magnitude of complex envelope of rogue wave records from Taitung Open Ocean buoy measured from 21:00h on 18 July 2015. The original signal of 600 s (time series **e**) is cut into different time length 75 s, 150 s, 300 s, 450 s, and sets the zero outside windows of duration as shown in **a** to **d**, respectively.

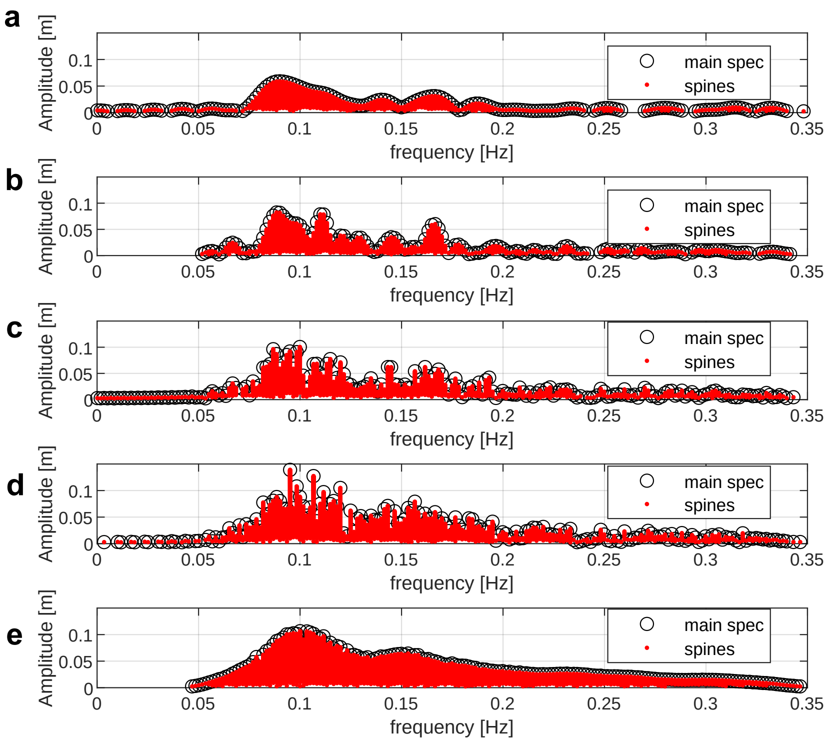

Figure 18: Additional example No. 5 for a stable-mode sample: Nonlinear spectra from **a** to **e** are obtained by NLSE-NFT with the magnitude of complex envelope in Figure 17 from **a** to **e**. The nonlinear spectrum of the time series in Figure 17e is classified as type 1: stable mode spectrum.

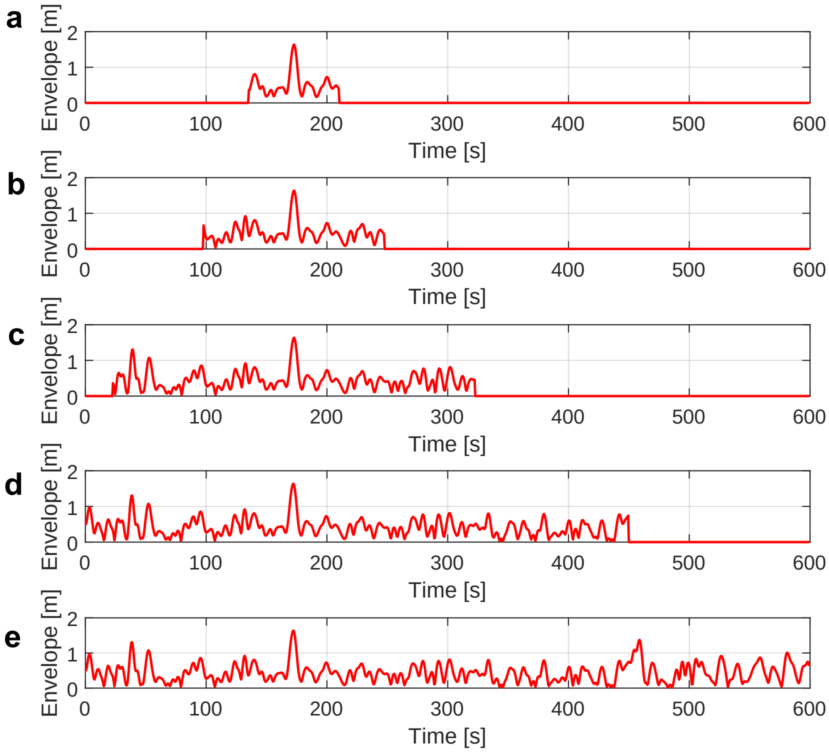

Figure 19: Additional example No. 1 for a small breather sample: Time series of magnitude of complex envelope of rogue wave records from Taitung Open Ocean buoy measured from 17:00h on 01 May 2012. The original signal of 600 s (time series **e**) is cut into different time length 75 s, 150 s, 300 s, 450 s, and sets the zero outside windows of duration as shown in **a** to **d**, respectively.

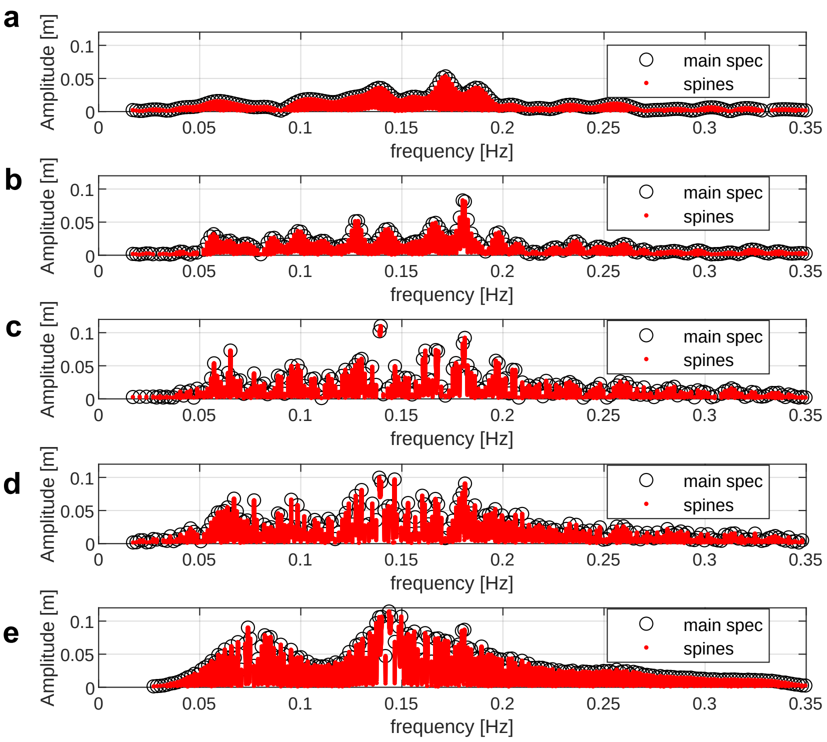

Figure 20: Additional example No. 1 for a small breather sample: Nonlinear spectra from **a** to **e** are obtained by NLSE-NFT with the magnitude of complex envelope in Figure 19 from **a** to **e**. The nonlinear spectrum of the time series in Figure 19e is classified as type 2: small breather spectrum.

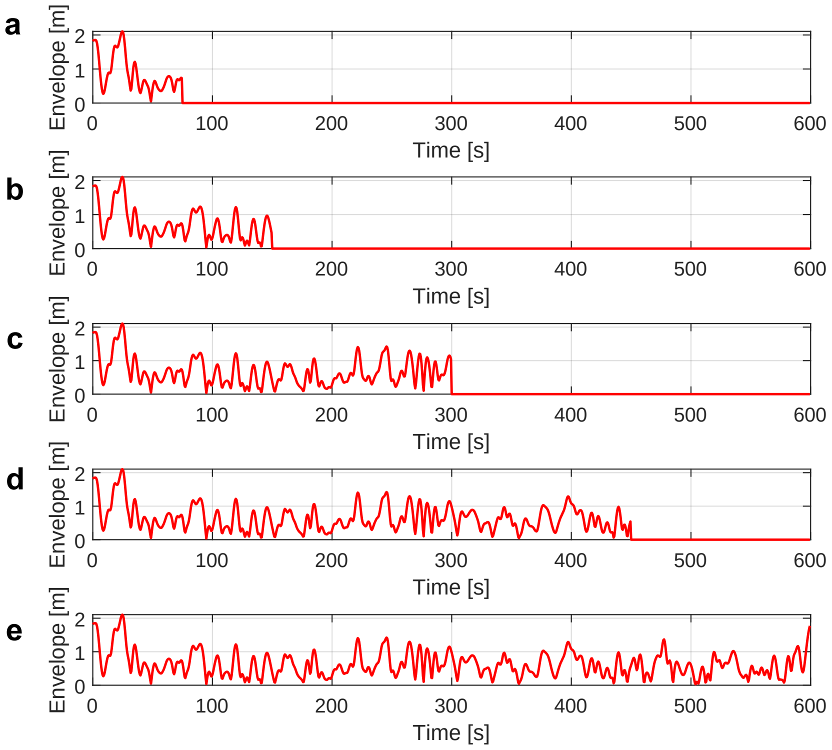

Figure 21: Additional example No. 2 for a small breather sample: Time series of magnitude of complex envelope of rogue wave records from Taitung Open Ocean buoy measured from 08:00h on 21 July 2014. The original signal of 600 s (time series **e**) is cut into different time length 75 s, 150 s, 300 s, 450 s, and sets the zero outside windows of duration as shown in **a** to **d**, respectively.

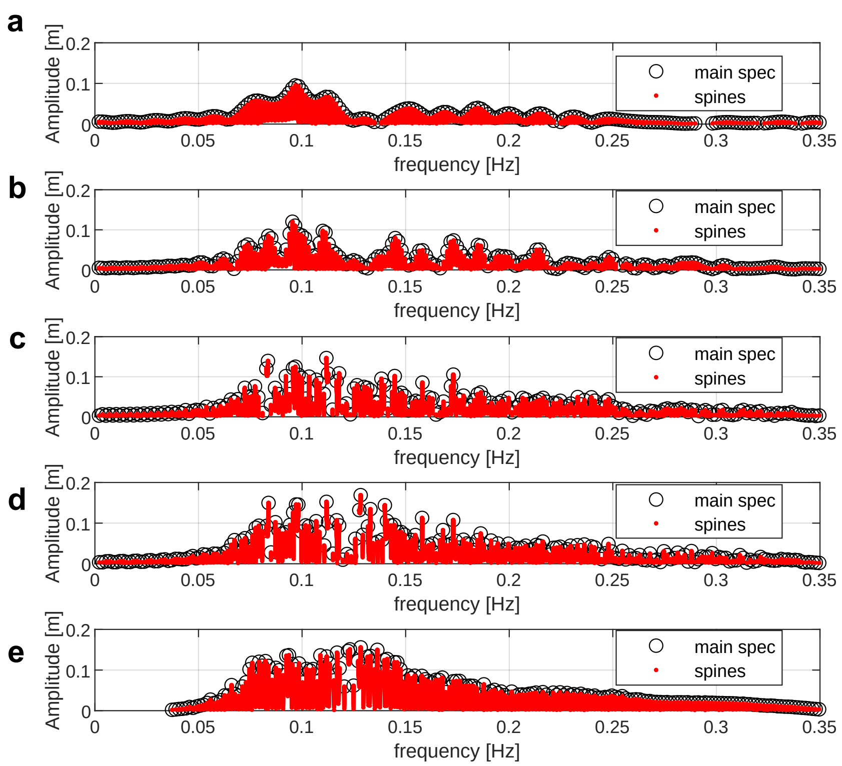

Figure 22: Additional example No. 2 for a small breather sample: Nonlinear spectra from **a** to **e** are obtained by NLSE-NFT with the magnitude of complex envelope in Figure 21 from **a** to **e**. The nonlinear spectrum of the time series in Figure 21e is classified as type 2: small breather spectrum.

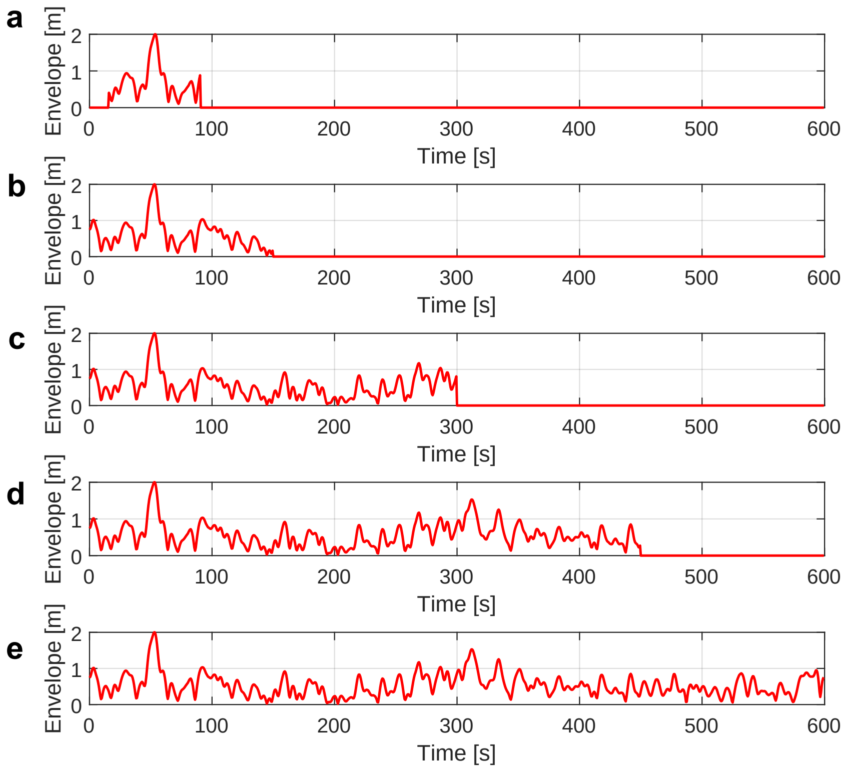

Figure 23: Additional example No. 3 for a small breather sample: Time series of magnitude of complex envelope of rogue wave records from Taitung Open Ocean buoy measured from 22:00h on 18 August 2014. The original signal of 600 s (time series **e**) is cut into different time length 75 s, 150 s, 300 s, 450 s, and sets the zero outside windows of duration as shown in **a** to **d**, respectively.

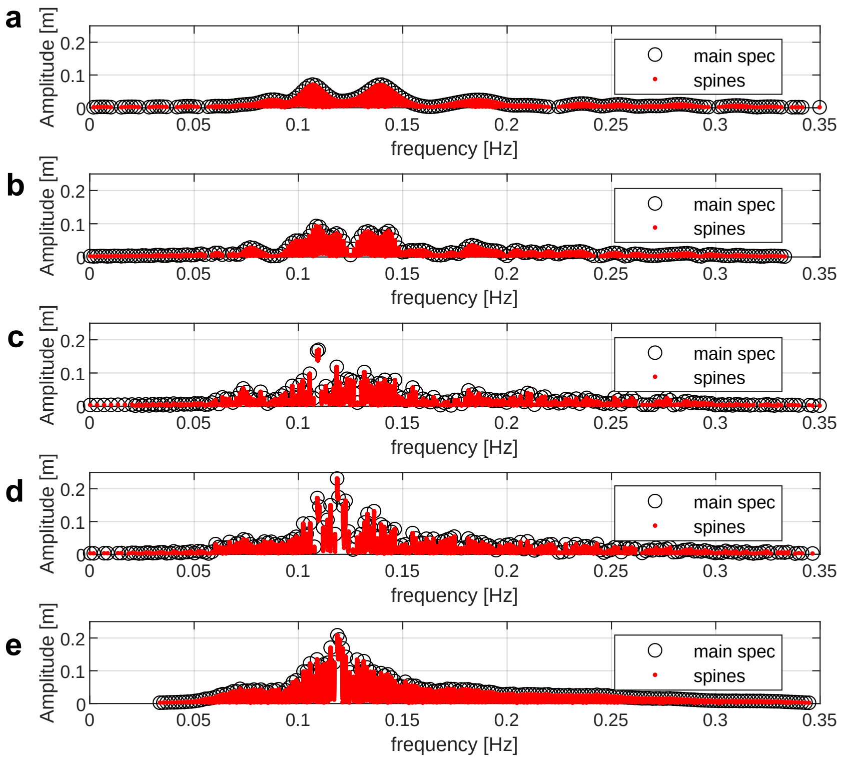

Figure 24: Additional example No. 3 for a small breather sample: Nonlinear spectra from **a** to **e** are obtained by NLSE-NFT with the magnitude of complex envelope in Figure 23 from **a** to **e**. The nonlinear spectrum of the time series in Figure 23e is classified as type 2: small breather spectrum.

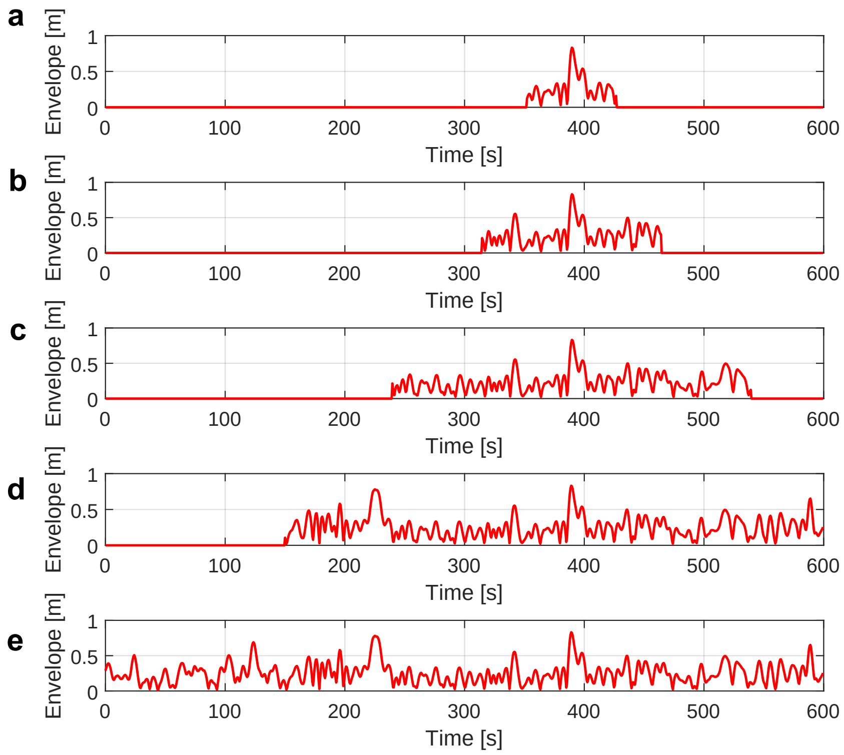

Figure 25: Additional example No. 4 for a small breather sample: Time series of magnitude of complex envelope of rogue wave records from Taitung Open Ocean buoy measured from 05:00h on 02 August 2015. The original signal of 600 s (time series **e**) is cut into different time length 75 s, 150 s, 300 s, 450 s, and sets the zero outside windows of duration as shown in **a** to **d**, respectively.

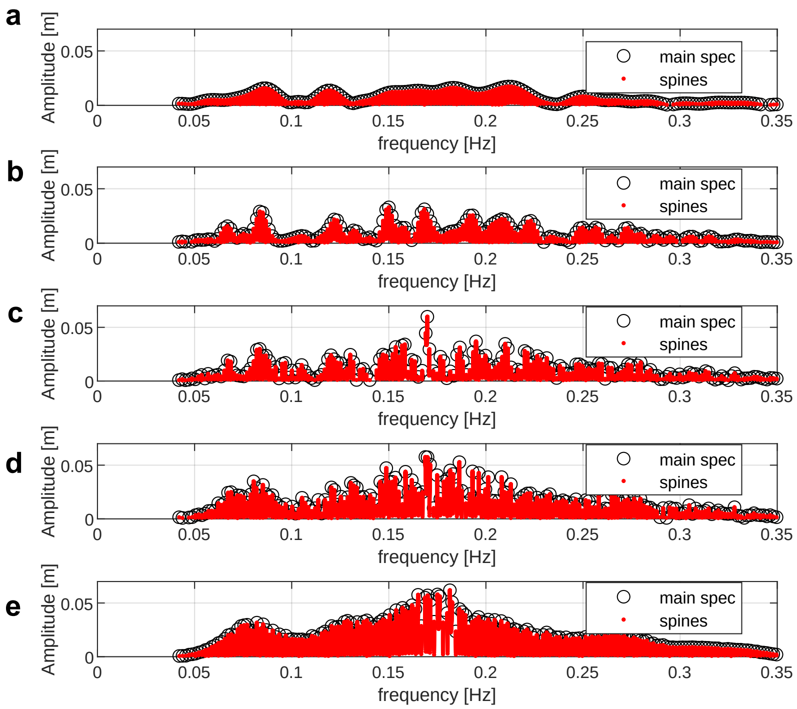

Figure 26: Additional example No. 4 for a small breather sample: Nonlinear spectra from **a** to **e** are obtained by NLSE-NFT with the magnitude of complex envelope in Figure 25 from **a** to **e**. The nonlinear spectrum of the time series in Figure 25e is classified as type 2: small breather spectrum.

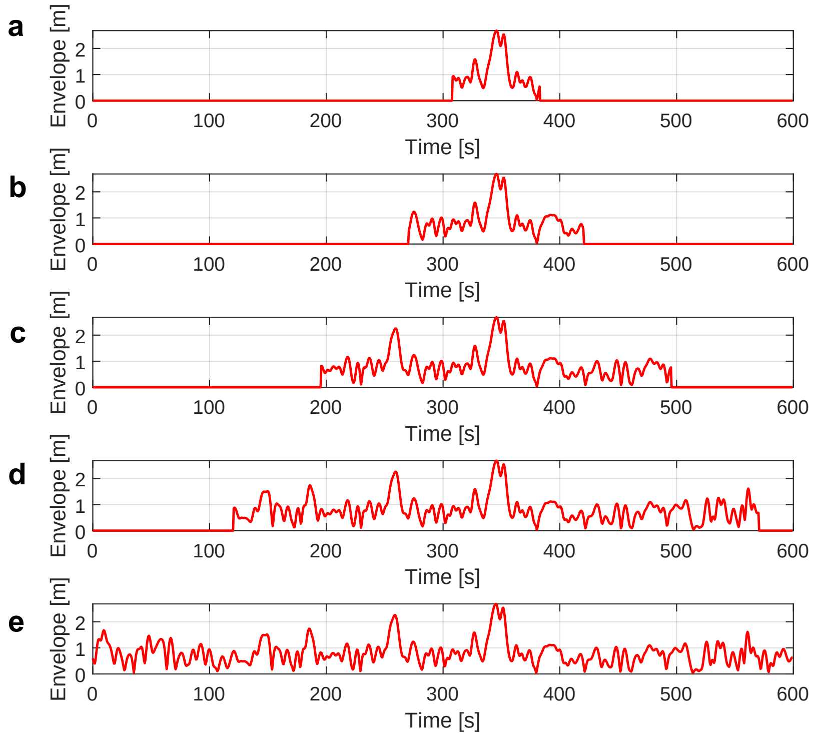

Figure 27: Additional example No. 5 for a small breather sample: Time series of magnitude of complex envelope of rogue wave records from Taitung Open Ocean buoy measured from 07:00h on 16 January 2016. The original signal of 600 s (time series **e**) is cut into different time length 75 s, 150 s, 300 s, 450 s, and sets the zero outside windows of duration as shown in **a** to **d**, respectively.

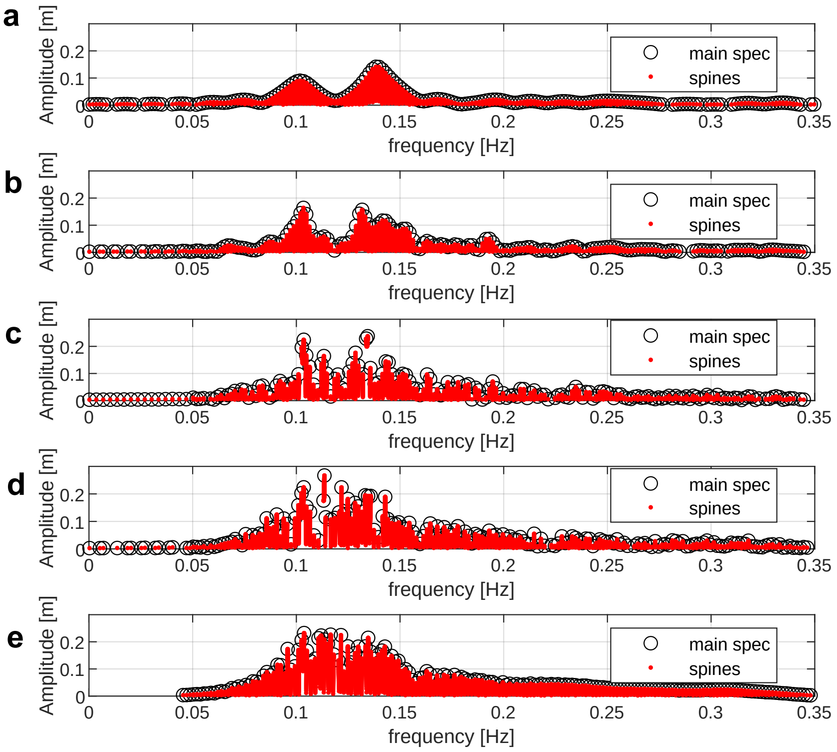

Figure 28: Additional example No. 5 for a small breather sample: Nonlinear spectra from **a** to **e** are obtained by NLSE-NFT with the magnitude of complex envelope in Figure 27 from **a** to **e**. The nonlinear spectrum of the time series in Figure 27e is classified as type 2: small breather spectrum.

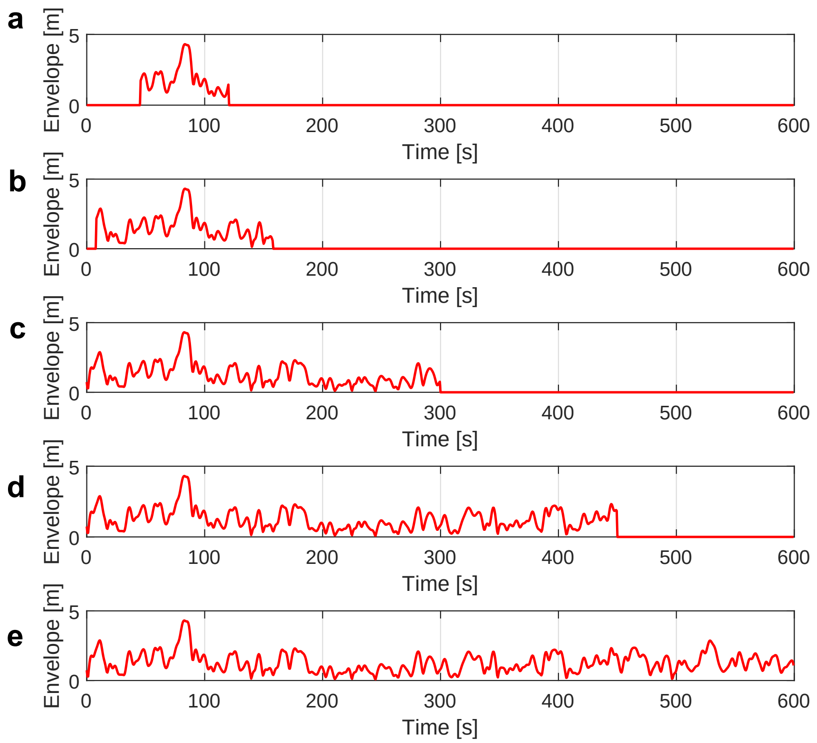

Figure 29: Additional example No. 1 for a large breather sample: Time series of magnitude of complex envelope of rogue wave records from Taitung Open Ocean buoy measured from 17:00h on 01 May 2012. The original signal of 600 s (time series **e**) is cut into different time length 75 s, 150 s, 300 s, 450 s, and sets the zero outside windows of duration as shown in **a** to **d**, respectively.

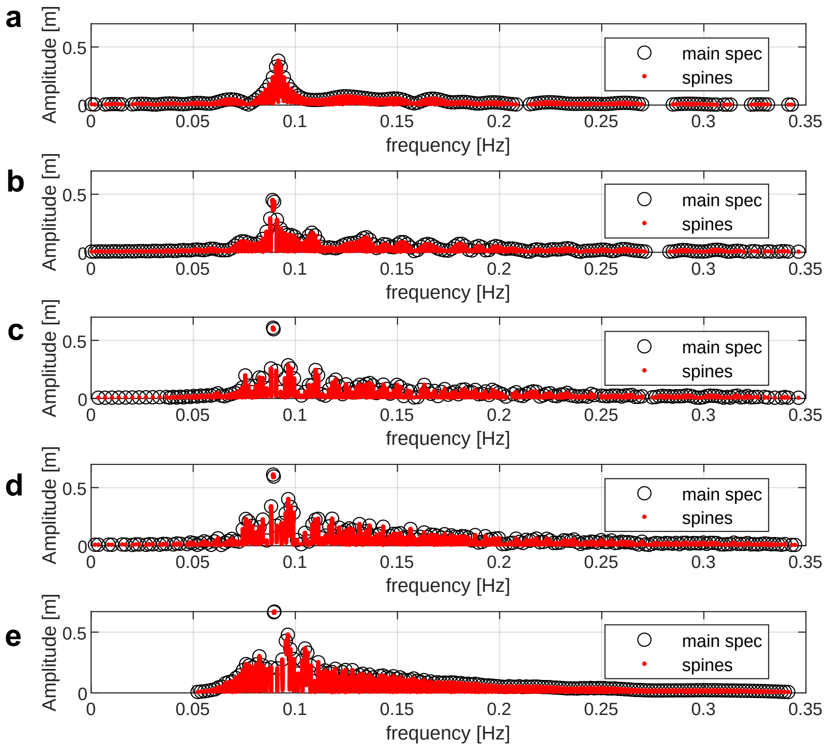

Figure 30: Additional example No. 1 for a large breather sample: Nonlinear spectra from **a** to **e** are obtained by NLSE-NFT with the magnitude of complex envelope in Figure 29 from **a** to **e**. The nonlinear spectrum of the time series in Figure 29e is classified as type 3: large breather spectrum.

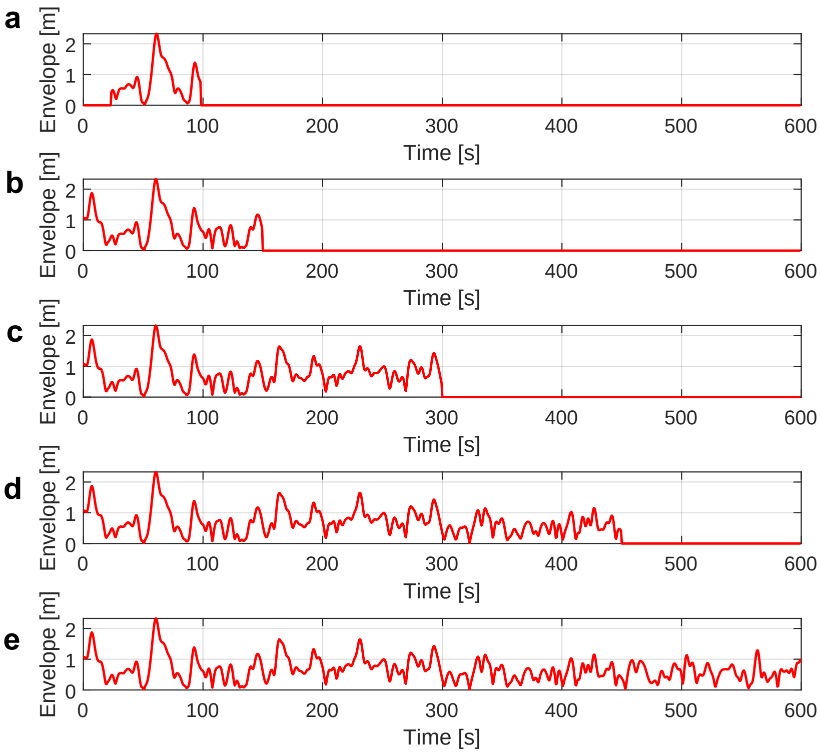

Figure 31: Additional example No. 2 for a large breather sample: Time series of magnitude of complex envelope of rogue wave records from Taitung Open Ocean buoy measured from 08:00h on 21 July 2014. The original signal of 600 s (time series **e**) is cut into different time length 75 s, 150 s, 300 s, 450 s, and sets the zero outside windows of duration as shown in **a** to **d**, respectively.

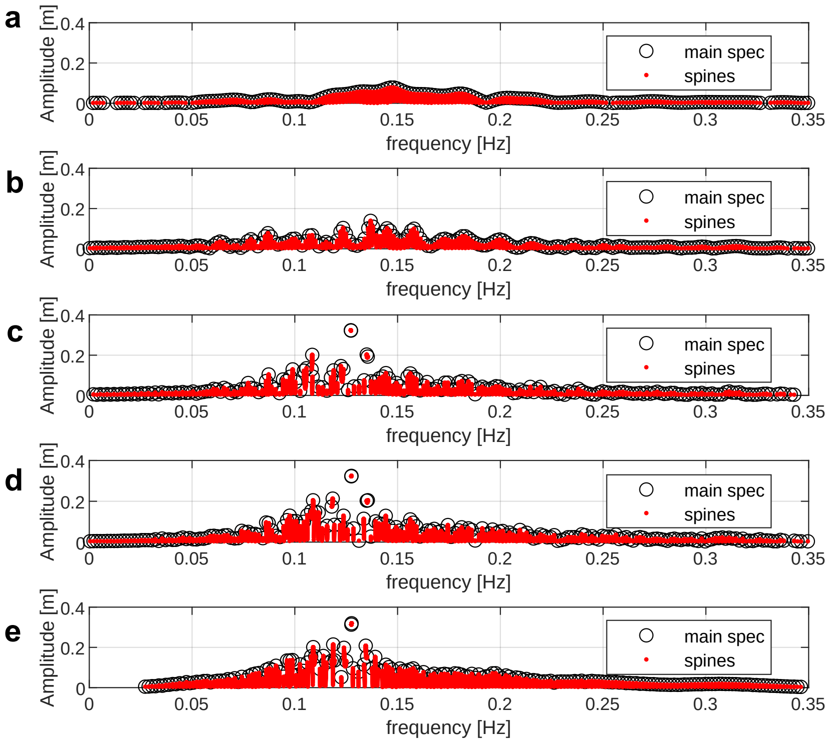

Figure 32: Additional example No. 2 for a large breather sample: Nonlinear spectra from **a** to **e** are obtained by NLSE-NFT with the magnitude of complex envelope in Figure 31 from **a** to **e**. The nonlinear spectrum of the time series in Figure 31e is classified as type 3: large breather spectrum.

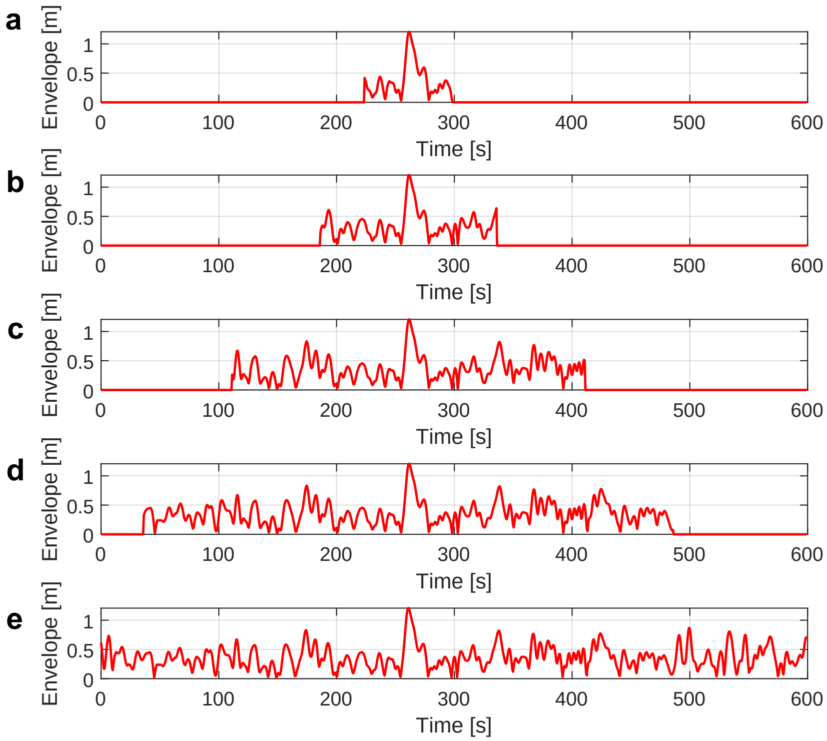

Figure 33: Additional example No. 3 for a large breather sample: Time series of magnitude of complex envelope of rogue wave records from Taitung Open Ocean buoy measured from 22:00h on 18 August 2014. The original signal of 600 s (time series **e**) is cut into different time length 75 s, 150 s, 300 s, 450 s, and sets the zero outside windows of duration as shown in **a** to **d**, respectively.

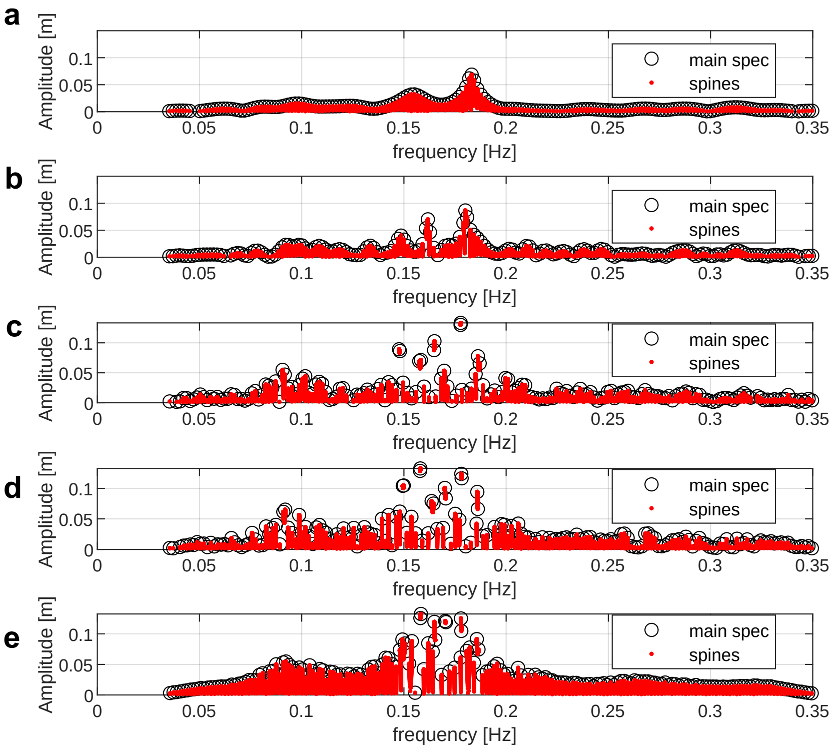

Figure 34: Additional example No. 3 for a large breather sample: Nonlinear spectra from **a** to **e** are obtained by NLSE-NFT with the magnitude of complex envelope in Figure 33 from **a** to **e**. The nonlinear spectrum of the time series in Figure 33e is classified as type 3: large breather spectrum.

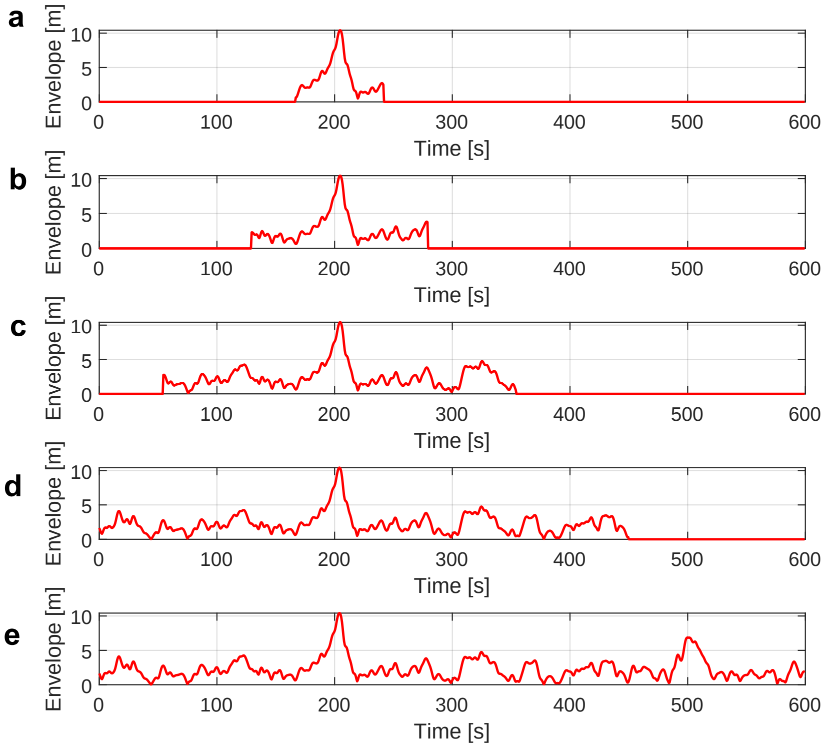

Figure 35: Additional example No. 4 for a large breather sample: Time series of magnitude of complex envelope of rogue wave records from Taitung Open Ocean buoy measured from 05:00h on 02 August 2015. The original signal of 600 s (time series **e**) is cut into different time length 75 s, 150 s, 300 s, 450 s, and sets the zero outside windows of duration as shown in **a** to **d**, respectively.

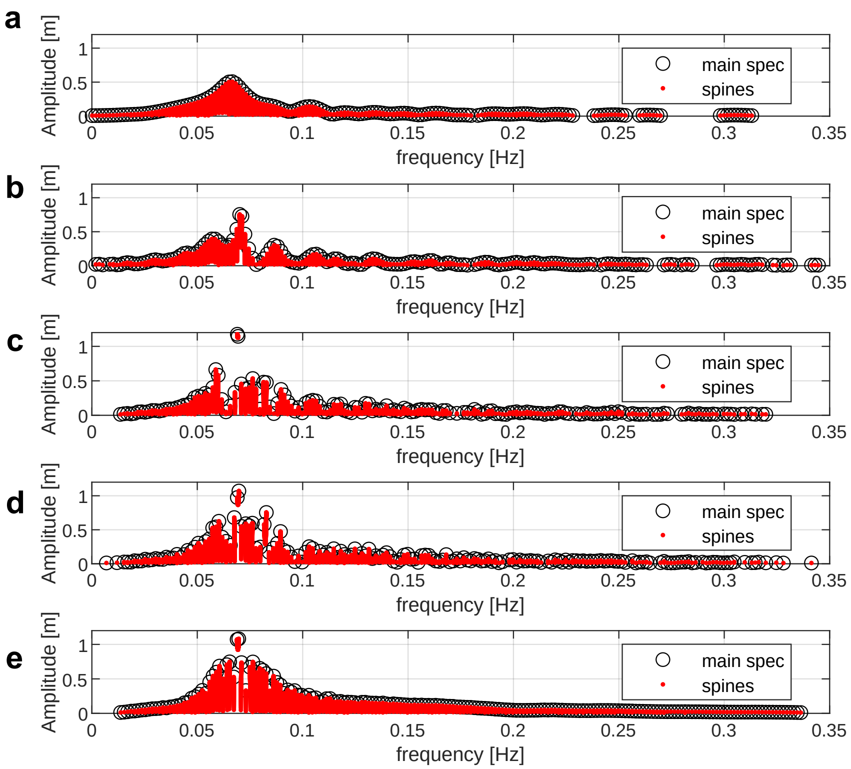

Figure 36: Additional example No. 4 for a large breather sample: Nonlinear spectra from **a** to **e** are obtained by NLSE-NFT with the magnitude of complex envelope in Figure 35 from **a** to **e**. The nonlinear spectrum of the time series in Figure 35e is classified as type 3: large breather spectrum.

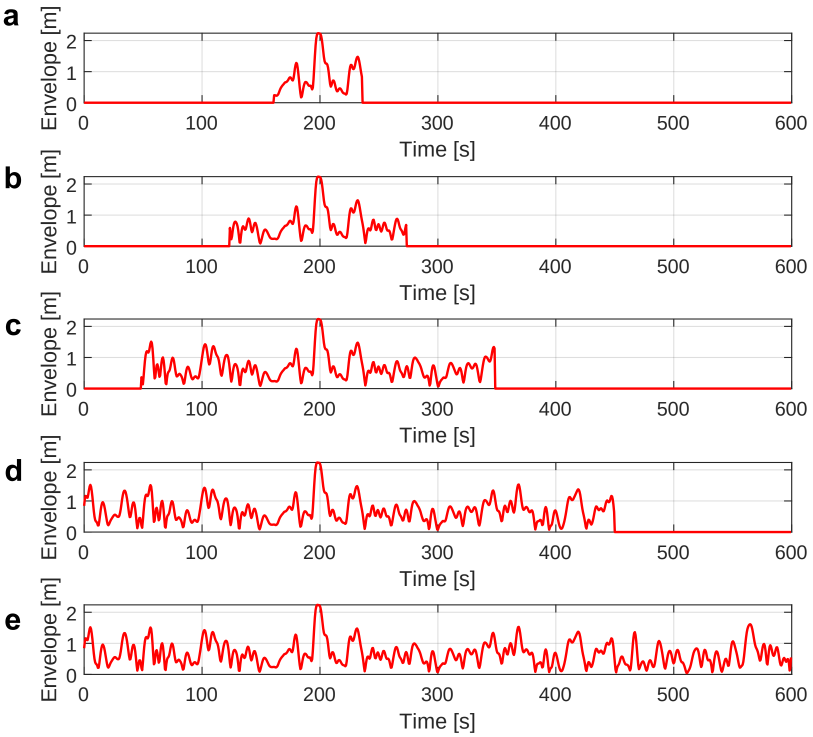

Figure 37: Additional example No. 5 for a large breather sample: Time series of magnitude of complex envelope of rogue wave records from Taitung Open Ocean buoy measured from 07:00h on 16 January 2016. The original signal of 600 s (time series **e**) is cut into different time length 75 s, 150 s, 300 s, 450 s, and sets the zero outside windows of duration as shown in **a** to **d**, respectively.

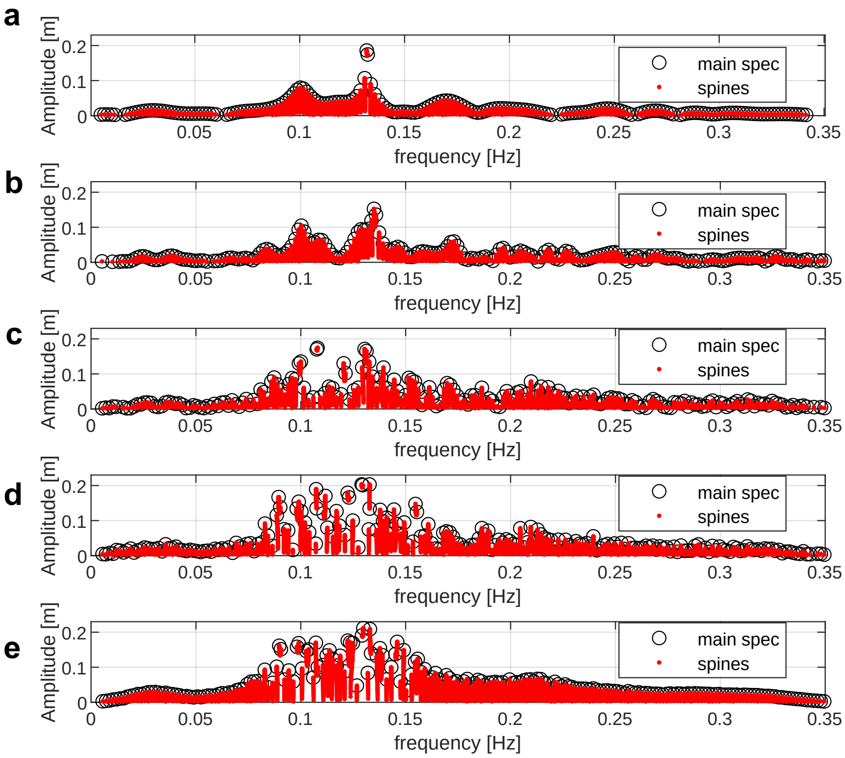

Figure 38: Additional example No. 5 for a large breather sample: Nonlinear spectra from **a** to **e** are obtained by NLSE-NFT with the magnitude of complex envelope in Figure 37 from **a** to **e**. The nonlinear spectrum of the time series in Figure 37e is classified as type 3: large breather spectrum.

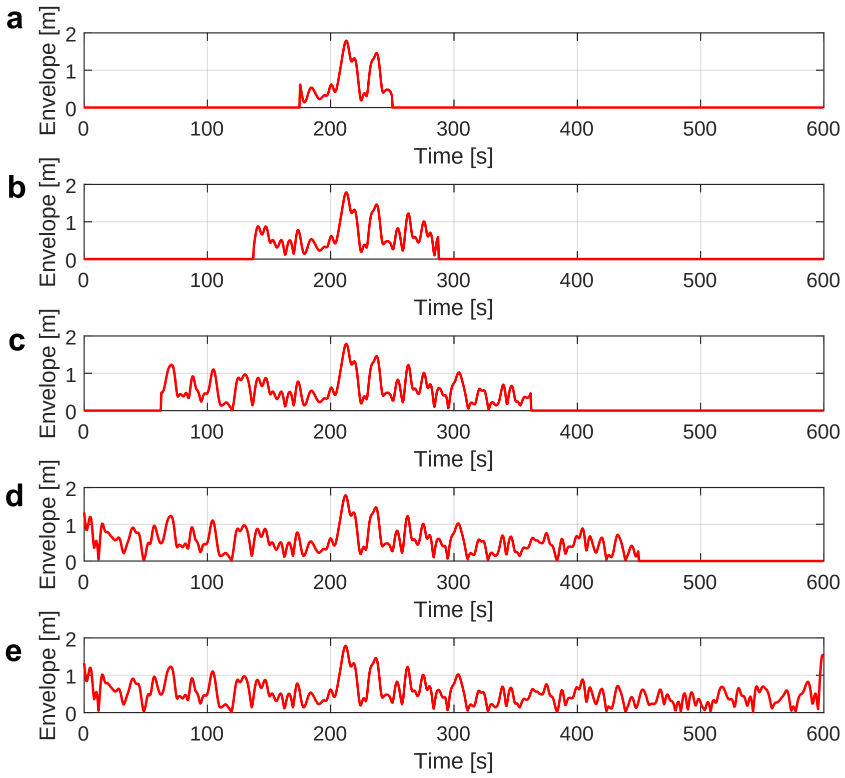

Figure 39: Additional example No. 1 for a soliton sample: Time series of magnitude of complex envelope of rogue wave records from Taitung Open Ocean buoy measured from 20:00h on 04 June 2006. The original signal of 600 s (time series **e**) is cut into different time length 75 s, 150 s, 300 s, 450 s, and sets the zero outside windows of duration as shown in **a** to **d**, respectively.

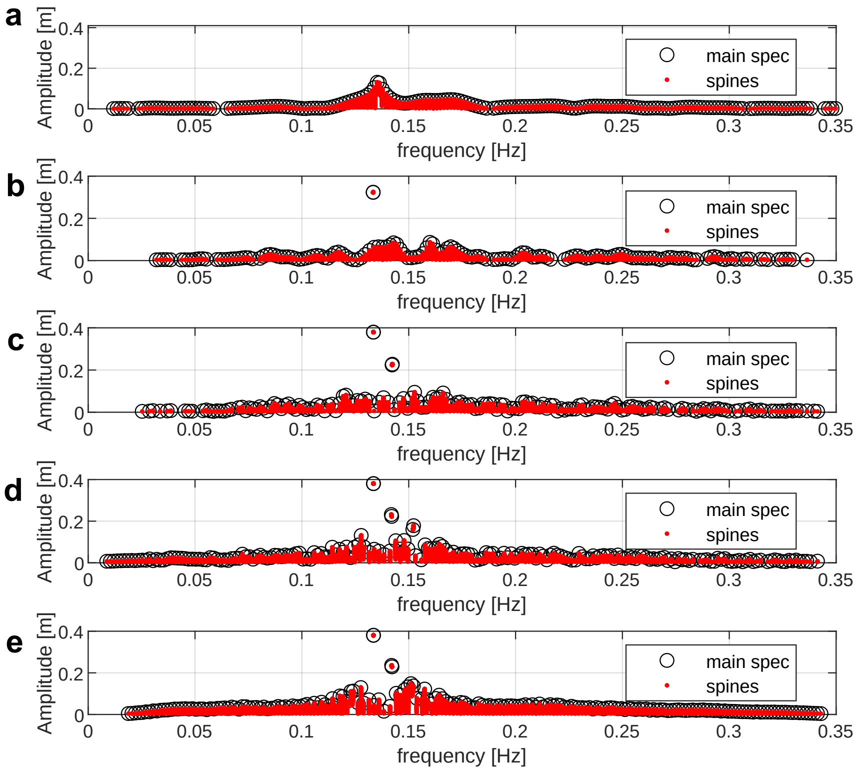

Figure 40: Additional example No. 1 for a soliton sample: Nonlinear spectra from **a** to **e** are obtained by NLSE-NFT with the magnitude of complex envelope in Figure 39 from **a** to **e**. The nonlinear spectrum of the time series in Figure 39e is classified as type 4: soliton spectrum.

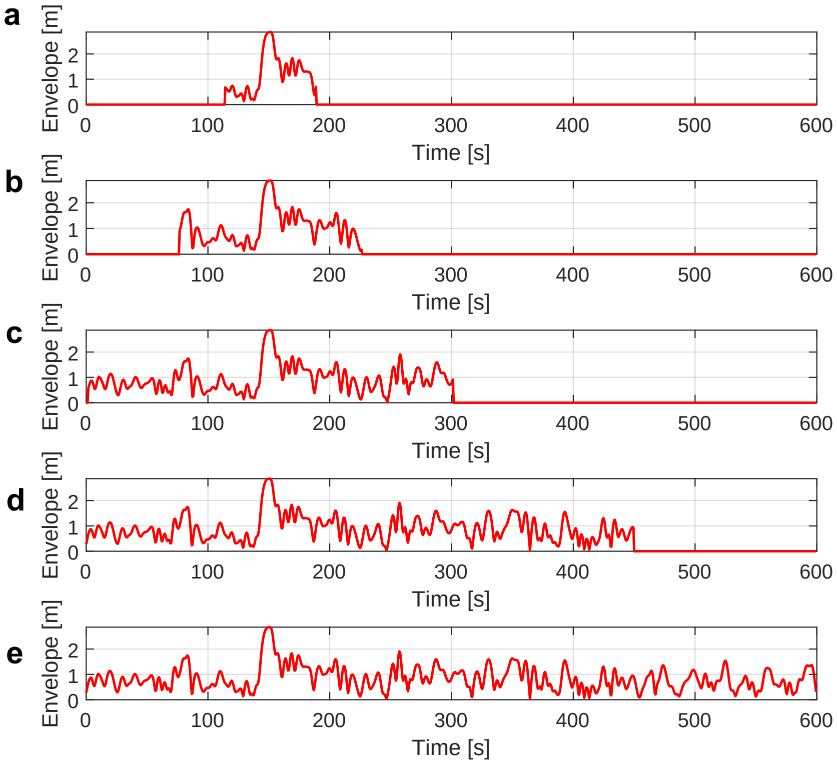

Figure 41: Additional example No. 2 for a soliton sample: Time series of magnitude of complex envelope of rogue wave records from Taitung Open Ocean buoy measured from 09:00h on 12 March 2012. The original signal of 600 s (time series **e**) is cut into different time length 75 s, 150 s, 300 s, 450 s, and sets the zero outside windows of duration as shown in **a** to **d**, respectively.

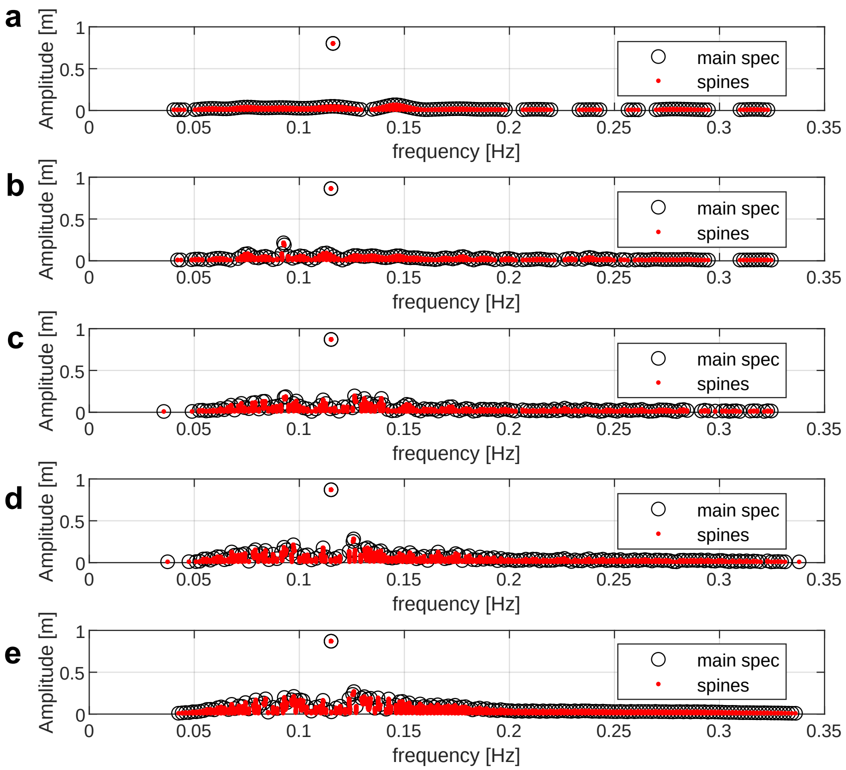

Figure 42: Additional example No. 2 for a soliton sample: Nonlinear spectra from **a** to **e** are obtained by NLSE-NFT with the magnitude of complex envelope in Figure 41 from **a** to **e**. The nonlinear spectrum of the time series in Figure 41e is classified as type 4: soliton spectrum.

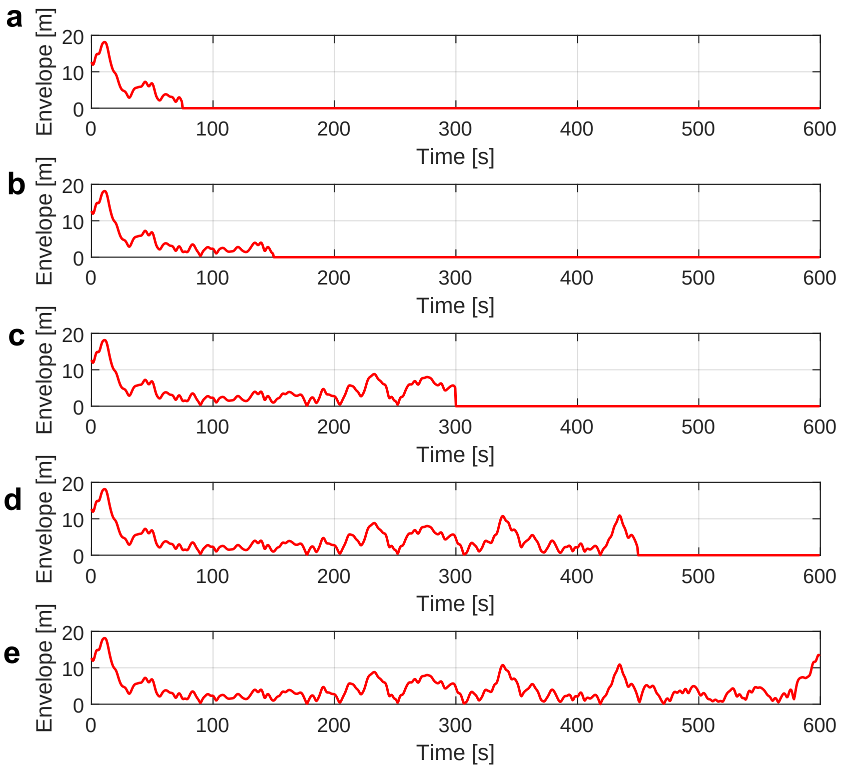

Figure 43: Additional example No. 3 for a soliton sample: Time series of magnitude of complex envelope of rogue wave records from Taitung Open Ocean buoy measured from 23:00h on 27 September 2012. The original signal of 600 s (time series **e**) is cut into different time length 75 s, 150 s, 300 s, 450 s, and sets the zero outside windows of duration as shown in **a** to **d**, respectively.

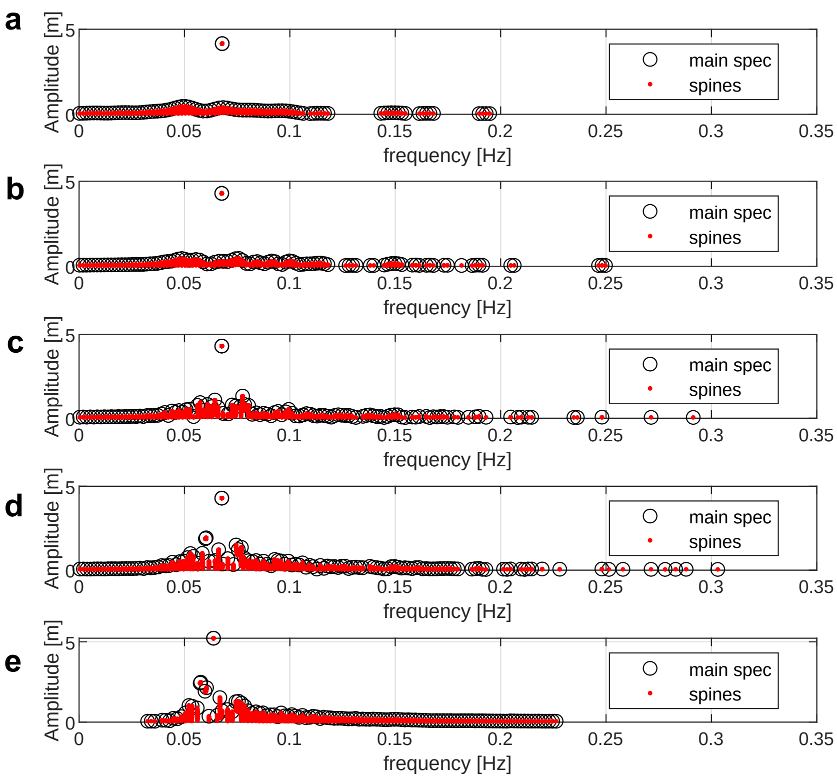

Figure 44: Additional example No. 3 for a soliton sample: Nonlinear spectra from **a** to **e** are obtained by NLSE-NFT with the magnitude of complex envelope in Figure 43 from **a** to **e**. The nonlinear spectrum of the time series in Figure 43e is classified as type 4: soliton spectrum.

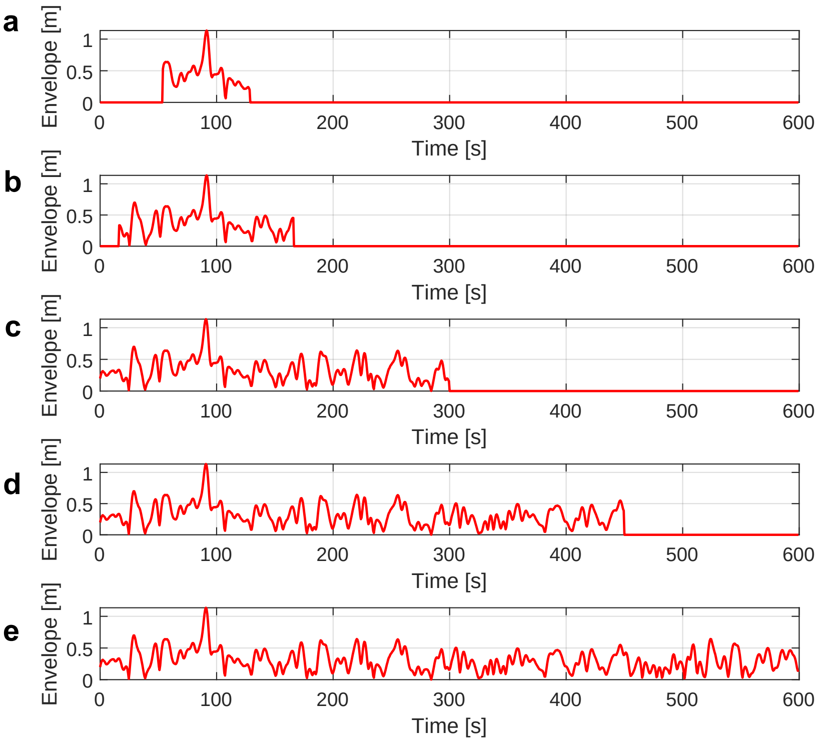

Figure 45: Additional example No. 4 for a soliton sample: Time series of magnitude of complex envelope of rogue wave records from Taitung Open Ocean buoy measured from 19:00h on 26 August 2014. The original signal of 600 s (time series **e**) is cut into different time length 75 s, 150 s, 300 s, 450 s, and sets the zero outside windows of duration as shown in **a** to **d**, respectively.

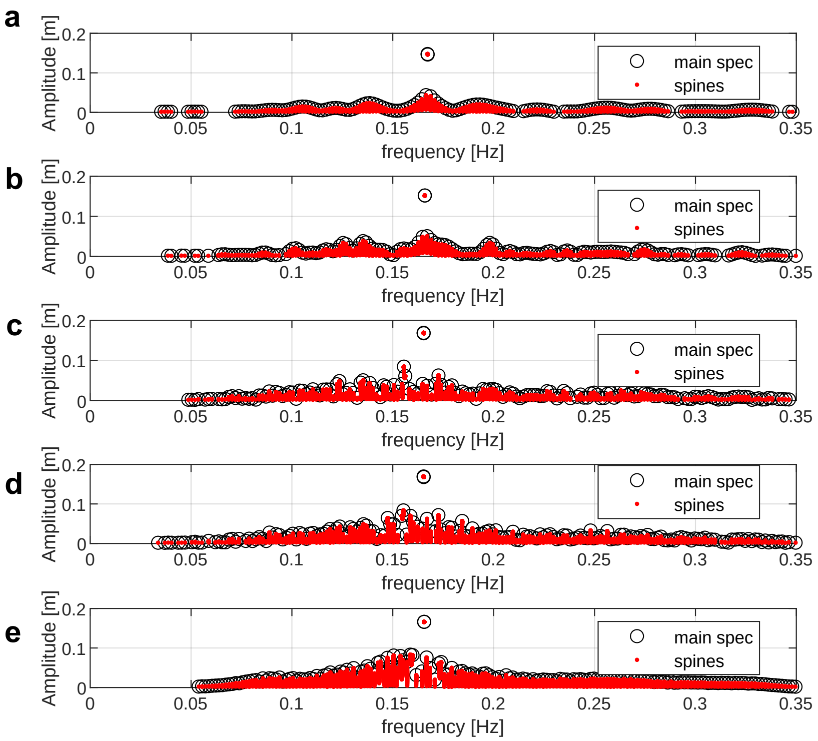

Figure 46: Additional example No. 4 for a soliton sample: Nonlinear spectra from **a** to **e** are obtained by NLSE-NFT with the magnitude of complex envelope in Figure 45 from **a** to **e**. The nonlinear spectrum of the time series in Figure 45e is classified as type 4: soliton spectrum.

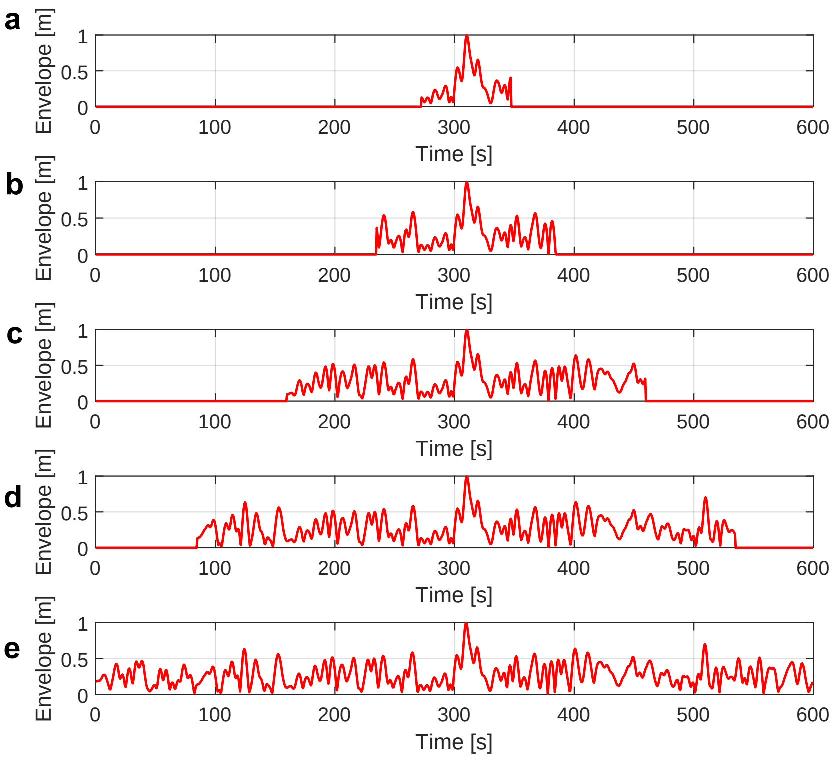

Figure 47: Additional example No. 5 for a soliton sample: Time series of magnitude of complex envelope of rogue wave records from Taitung Open Ocean buoy measured from 15:00h on 02 February 2015. The original signal of 600 s (time series **e**) is cut into different time length 75 s, 150 s, 300 s, 450 s, and sets the zero outside windows of duration as shown in **a** to **d**, respectively.

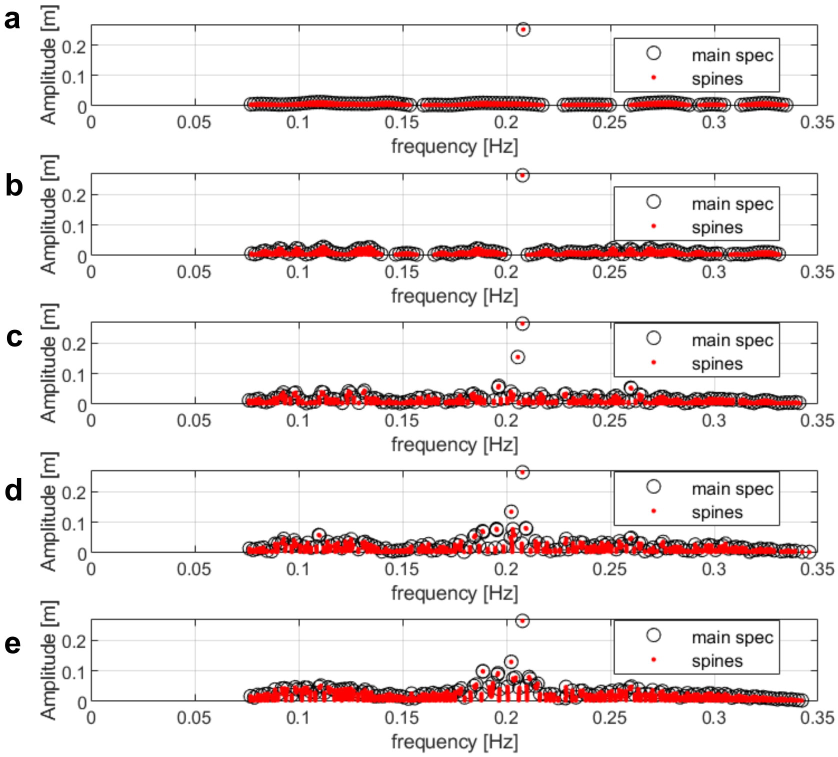

Figure 48: Additional example No. 5 for a soliton sample: Nonlinear spectra from **a** to **e** are obtained by NLSE-NFT with the magnitude of complex envelope in Figure 47 from **a** to **e**. The nonlinear spectrum of the time series in Figure 47e is classified as type 4: soliton spectrum.
