# Supplementary material for: Nonlinear Fourier classification of 663 rogue waves measured in the Philippine Sea
Source: PLoS One. 2024 May 14;19(5):e0301709. doi: 10.1371/journal.pone.0301709 (PMC11093313; doi:10.1371/journal.pone.0301709)
Supplement: Attachment — Submitted filename: Report.pdf [file pone.0301709.s002.pdf]

Review on the manuscript PONE-D-23-29589

"Nonlinear Fourier Classification of 663 Rogue Waves Measured in the Philippine Sea"
by Y.-C. Lee, M. Bruhl, D.-J. Doong, S. Wahls
submitted for publication in PLOS ONE

In this work, the Inverse Scattering Technique (IST) is used to analyze a large dataset of instrumental in-situ measurements in order to reveal possible correlations between the nonlinear spectral data and rogue wave events. Since the data correspond to deep-water conditions, the integrable nonlinear Schrodinger equation (NLSE) is used as the leading-order approximation. The IST is employed as a tool for a signal analysis rather than an approach for exact solution of the NLSE. This idea is not new, see e.g. Ref. 44, though its significance to the ocean wave data analysis remains so far unclear and is disputed. The IST is a mathematically complicated method; several competing approaches have been proposed in the literature to apply to hydrodynamic data. Thus, it requires time to be accepted by oceanographers and to understand the potential of this approach. The present manuscript brings the underpinning mathematical theory to a reader in a most friendly way and presents results of analysis of a large amount of in-situ deep-water data for the first time. I believe that the paper deserves publication in a top-level journal like PLOS ONE. At the same time, the manuscript is not ready for publication in its present form. It requires **major revision** as pointed out below.

It is mentioned in the introduction than the BFI parameter was introduced in theoretical works in order to quantify the effect of modulational instability. This parameter is already integrated into the ECMWF warning system, though seemingly with no noticeable success. Indeed, values of BFI may give a rough idea about the strength of nonlinear wave self-modulation and possible parameters of breathers. That is why it is worthy to show the values of BFI of the measured waves and to discuss them in relation to the performed classification of cases as 'stable', 'small breather', etc. Besides the 1D version of BFI, the authors have the information sufficient to calculate 2D BFIs following e.g. [N. Mori, M. Onorato, P.A.E.M. Janssen, J. Phys. Oceanogr. 41, 1484-1497 (2011) or A. Ribal et al., J. Fluid Mech. 719, 314-344 (2013)]. How do these values correlate with the expectation of the Benjamin – Feir instability? It seems to me that the broad angular spectrum (see Fig. 3d,h) should make manifestations of the Benjamin – Feir instability absolutely impossible. This issue should be discussed.

<u>Other remarks.</u>

1. The first sentence of the abstract contains the word "wave(s)" 5 times. Please, paraphrase.
2. The statement "*this is the first time that it is systematically applied to a large real-world dataset*" in the abstract (and also in lines 544-546) is not completely true. A large dataset was analyzed using the IST-based approach in Ref. 53 (coauthored by two of the authors of this manuscript), though the shallow water case using the Korteweg – de Vrier framework was considered in that paper.
3. Line 16: "*See [25] for a discussion of further literature*" The reference for 'further literature', dated 2000, looks strange.
4. Line 67. The sentence "*Most parts of the nonlinear spectrum are even constants of motion, i.e., they do not change at all during propagation with respect to the NLSE*" is not clear. Do 'most parts' correspond to the 'main spectrum' mentioned in line 163?

5. Line 83. The breather-like solutions survive in opposite wave trains too, see the relevant reference [Y. He, A. Slunyaev, N. Mori, A. Chabchoub, Phys. Rev. Lett. 129, 144502 (2022)].
6. Eq. (2). According to the text just below, it should be E_k not *Ek*. The parameter n is not introduced.
7. Eq. (6). What is N?
8. Fig. 3. I guess that rogue waves with heights below some level were not processed. May it be the reason why the maxima of the histograms for $H_{1/3}$ and $H_{\max}$ correspond to the minimum values of $H_{1/3}$ and $H_{\max}$ for usual series, but not for rogue wave series?

   What is N? I suspect, the total number of rogue wave samples should be much smaller than the number of ordinary wave samples, but do not see this in the figure.
9. Line 209. The Hilbert transform is known to be an inaccurate method for constructing envelopes of nonlinear Stokes waves due to the phase-locked nonlinear harmonics. Please comment on that.
10. Sec. "Data normalization of ocean waves in finite depth".

    I believe, the depth of 5 km well corresponds to the deep water condition. It is straightforward to estimate that the dimensionless depth parameters are $k_p h = 200$ for the most typical period $T_p = 10$ s and $k_p h = 50$ for the longest period according to Fig. 3, $T_p = 20$ s. Therefore there is no need to introduce the finite-depth theory, the water is effectively infinitely deep.

    In Eq. (11) the 'nonlinear parameter' ρ should contain ν (\nu) not v (v). The relations are just linear scaling of variables which reduces Eq. (7) to Eq. (1). Therefore the sentence "*By applying the multi-dimensional chain rule and substituting* $A = \rho^{-1}$ *in the temporal NLSE, one obtains the normalized temporal NLSE (1)*" is not understood at all.

    To conclude, this section may be of a few lines length, no longer.
11. The purpose why the parameter ρ is introduced is not clear (it seems to be unused below?). The relations (11) do not produce non-dimensional variables u, X, T, the variables are still dimensional. In particular, the parameter ρ is dimensional, $m^{-1}c^{-1}$, if the envelope $A(x,t)$ describes the surface displacement, as usually. Therefore, the role of the transformation (11) is not clear.
12. Sec. "Rogue wave criterion and its formation" is not well written. It is discussed several times within a few lines that the down-crossing method is used, that $H_{1/3}$ is the significant wave height, etc.
13. Line 259. Right before, the case of an envelope soliton in Fig. 4b is discussed, which is known to be a stable structure. Then, the sentence "*Arbitrarily small perturbations can free the trapped auxiliary spectrum points ... so that the solution is unstable to perturbations*" is not clear.
14. Lines 267-276. Honestly, I understood almost nothing. There are many words in comas, what probably should mean some lack of rigor in the description. All together, this looks descriptive and uncertain. The paragraph above (lines 248-266) is a bit better.

    The eigenvalues λ in Fig. 4 come 'from the sky'.

    I see that the topic is mathematically complicated and cannot be fully explained in a few lines, but in the present form the description does not give any idea of origins of the presented details of the theory (see also some more critical remarks below).
15. Line 279. I do not understand how the degenerate and non-degenerate points may be distinguished in Fig. 4b.

16. Line 285. The expression for the soliton solution is obviously wrong. Should be "sech(T)?"
17. Line 287. The description "*The two points are however distinct. Since they are very close, the spine cannot be seen in the Fig. 4d*" is unclear. In Fig. 4b the points are $\pm i$ (not very close), though $\pm i/2$ in Fig. 4d are 'too close' ?..

    A related question arises when the cases 3 and 4 of the nonlinear spectrum classification are discussed: a quantitative difference between a 'large amplitude breather' and a 'soliton' is not presented.
18. Line 289. The sentence "*The well-known breather solutions of the NLSE with periodic boundary conditions include Akhmediev breather, Peregrine solitons, Kuznetsov-Ma solitons*" is inaccurate. Either Akhmediev of Kuznetsov breather is periodic along the chosen variable. The Peregrine breather is not periodic at all.
19. Line 294. What are α_3, ρ, η??? In addition, α_1 and α_2 appears a few lines below, which are not explained at all…
20. Fig. 4. Please add the legend for the main points and spines similar to as done in Fig. 6.
21. Fig. 6. Coefficients of the NLSE are written in terms of the carrier wavenumber and frequency, while the wave spectrum is assumed to be narrow. The frequency band in Fig. 6 is a few octaves. How is it possible? How k_0 and ω_0 were calculated? They are key parameters of the NLSE which specify whether wave groups are stable or not. The applied method imposes periodic boundary conditions, though they are not. How do you make the data periodic?
22. Fig. 7. Please make the caption more precise: "*…based on the four types of* ***nonlinear*** *spectra.*"
23. Line 403. Based on the preceding discussion, I assume that the sentence "*This suggests that the existence of unstable modes in nonlinear spectra is related to large waves in real ocean…*" is not fully representative. It is correct for a given T_p, though the authors demonstrate a strong dependence on T_p as well. I believe that the wave steepness $\varepsilon \propto kH \propto H/T^2$ may be an appropriate governing parameter for the cases of 'solitons' and 'large amplitude breathers' in Fig. 7a,b. It gives a square-root dependence $T \sim H^{1/2}$ which seems to correspond to the observational data (see 'solitons' in Fig. 7a,b).

    This remark also influences the statements in lines 507-508 and 539-541.
24. Line 419. The text "*within our the limits of our data set*" is obviously corrupted.
25. Line 420. Please, give explicitly the range of directional spreads and the method how the angular spectrum width was calculated.
26. Line 425. What does "*the largest unstable modes*" mean? Is it about the class 4?
27. Line 429. Here and in other examples the information about $H_{1/3}$ and $H_{\max}$ is given but not about T_p. Please indicate T_p.
28. Line 431. It is not clear from the description that all the frames are centered at the extreme wave event at $t \approx 218$ s. The frames in Fig.9a may be seen only from a monitor when the page is magnified. Please improve the representation.
29. Line 475. The sentence "*The finding that for soliton type spectra, the soliton is found in the rogue wave has been confirmed in all but one cases*" is repeated twice.
30. Line 484. The sentence "*We therefore relate the amplitude of the largest nonlinear mode to the maximum wave height of the time series with rogue waves in a scatter plot in Fig. 10*" is not accurate: maximum **crest** height is plotted in Fig. 10, not the maximum height.

    In fact, there is more sense to plot amplitudes of the maximum nonlinear modes in Fig. 10 versus **maximum height** (not crest height). The wave crest height of nonlinear

Stokes waves may be significantly larger than the half-height of the wave due to the phase-locked bound waves. This effect is not taken into account by the authors when the complex envelope is obtained from the surface displacement. The second-order bound waves are effectively eliminated in the wave height quantity.

31. Line 493. Please paraphrase "*higher crest heights*".
