# Supplementary material for: Nonlinear Fourier classification of 663 rogue waves measured in the Philippine Sea
Source: PLoS One. 2024 May 14;19(5):e0301709. doi: 10.1371/journal.pone.0301709 (PMC11093313; doi:10.1371/journal.pone.0301709)
Supplement: Attachment — Submitted filename: PLOS_ONE_authors_response.pdf [file pone.0301709.s003.pdf]

Authors Response to the Reviewer Comments

Y.-C. Lee, M. Brühl, D.J. Doong and S. Wahls

Dear Professor Malomed,

Thank you for offering us the opportunity to submit a revised draft of our manuscript "Nonlinear Fourier Classification of 663 Rogue Waves Measured in the Philippine Sea" to PLOS ONE. We appreciate the time and effort that you and the reviewer have dedicated to providing feedback on our manuscript, which we have revised significantly. Please find our point-by-point response to the reviewers' comments and concerns below.

Reply to Reviewer 1

In this work, the Inverse Scattering Technique (IST) is used to analyze a large dataset of instrumental in-situ measurements in order to reveal possible correlations between the nonlinear spectral data and rogue wave events. Since the data correspond to deep-water conditions, the integrable nonlinear Schrodinger equation (NLSE) is used as the leading-order approximation. The IST is employed as a tool for a signal analysis rather than an approach for exact solution of the NLSE. This idea is not new, see e.g. Ref. 44, though its significance to the ocean wave data analysis remains so far unclear and is disputed. The IST is a mathematically complicated method; several competing approaches have been proposed in the literature to apply to hydrodynamic data. Thus, it requires time to be accepted by oceanographers and to understand the potential of this approach. The present manuscript brings the underpinning mathematical theory to a reader in a most friendly way and presents results of analysis of a large amount of in-situ deep-water data for the first time. I believe that the paper deserves publication in a top-level journal like PLOS ONE. At the same time, the manuscript is not ready for publication in its present form. It requires major revision as pointed out below.

Reply: Thank you for your comment. The nonlinear Fourier transform (NFT), also known as the inverse scattering transform (IST), has indeed already been used as a signal processing tool in various areas, including oceanic engineering. Although the theory has existed for a long time, there are not many examples of typical nonlinear spectra of ocean waves in the literature.

We appreciate the reviewer's agreement that our paper could be a valuable

publication for PLOS ONE. We have revised the paper based on the reviewer's comments to provide a better demonstration and understanding of our results.

It is mentioned in the introduction than the BFI parameter was introduced in theoretical works in order to quantify the effect of modulational instability. This parameter is already integrated into the ECMWF warning system, though seemingly with no noticeable success. Indeed, values of BFI may give a rough idea about the strength of nonlinear wave self-modulation and possible parameters of breathers. That is why it is worthy to show the values of BFI of the measured waves and to discuss them in relation to the performed classification of cases as 'stable', 'small breather', etc. Besides the 1D version of BFI, the authors have the information sufficient to calculate 2D BFIs following e.g. [N. Mori, M. Onorato, P.A.E.M. Janssen, J. Phys. Oceanogr. 41, 1484-1497 (2011) or A. Ribal et al., J. Fluid Mech. 719, 314-344 (2013)]. How do these values correlate with the expectation of the Benjamin – Feir instability? It seems to me that the broad angular spectrum (see Fig. 3d,h) should make manifestations of the Benjamin – Feir instability absolutely impossible. This issue should be discussed.

Reply: This is an excellent suggestion. We have added definitions of both the one-dimensional and two dimensional BFI parameter to the text in the subsection "Rogue waves and sea state parameters". A plot showing their distribution over wave heights and a table with mean BFIs for the various types have been added to the subsection "Data analysis of wave samples with four types of nonlinear spectra: stable, small and large breather, soliton". These additional results are interpreted in the section "Summary and discussion".

1. *The first sentence of the abstract contains the word "wave(s)" 5 times. Please, paraphrase.*

Reply: Thanks for the hint. We have revised the sentence to "Rogue waves are sudden and extreme occurrences, with heights that exceed twice the significant wave height of their neighboring waves.".

2. *The statement "this is the first time that it is systematically applied to a large realworld dataset" in the abstract (and also in lines 544-546) is not completely true. A large dataset was analyzed using the IST-based approach in Ref. 53 (coauthored by two of the authors of this manuscript), though the shallow water case using the Korteweg – de Vrier framework was considered in that paper.*

Reply: The claim in the abstract was the *NLSE*-NFT is used to analyze a large number of rogue waves for the first time, which to the best of our knowledge is correct. In Teutsch et al. [1], the KdV-NLFT was used instead. To make this difference clearer, we now explicitly point out that we are considering deep wave rogue waves. The sentence has been revised to "While the NLSE-NFT has been used to analyze rogue waves before, this is the first time that it is systematically applied to a large real-world dataset of deep-water rogue waves."

3. *Line 16: "See [25] for a discussion of further literature" The reference for*

'further literature', dated 2000, looks strange

Reply: Thank you. We have added additional references.

4. *The sentence "Most parts of the nonlinear spectrum are even constants of motion, i.e., they do not change at all during propagation with respect to the NLSE" is not clear. Do 'most parts' correspond to the 'main spectrum' mentioned in line 163?*

Reply: This sentence was indeed referring to the main spectrum. It has been revised to make this clearer.

We also added a sentence at the end of the "Operation of Periodic NLSE-NFT operation" subsection to point out that the spines are invariant as well.

5. *Line 83. The breather-like solutions survive in opposite wave trains too, see the relevant reference [Y. He, A. Slunyaev, N. Mori, A. Chabchoub, Phys. Rev. Lett. 129, 144502 (2022)*

Reply: Thank you for the the reference. We have integrated it into the revised version.

6. *Eq. (2). According to the text just below, it should be E_k not Ek. The parameter n is not introduced.*

Reply: Thanks for these comments. The typo with the E_k has been fixed.

The parameter n, which has been renamed to g in the revised manuscript because of a notational conflict, determines the number of hyperelliptic modes. For historical reasons, it is known as the number of "gaps" (for the KdV-NLFT, the spectral problem has gaps between bands of spectrum, the number of which is finite for finite gap solutions). Another common name is the "genus" because it corresponds to the genus of the Riemannian surface that is associated with the main spectrum. While g does not have to be finite, there are only few results in the literature for infinite-gap solutions. In a way, finite-gap solution can be considered a nonlinear analog of a conventional Fourier series with only finitely many terms. A proper discussion of these issues would become quite lengthy and distract the reader, but we added the following sentence: "The number g of hyperelliptic modes, which is also known as the number of "gaps" or the "genus" in the literature, is assumed to be finite for mathematical reasons."

7. *Eq. (6). What is N?*

Reply: This should have again been the number of gaps g. We apologize for the typo. As already mentioned, we changed to number of gaps to g throughout the document to avoid confusion between n and $\boldsymbol{n}$.

8. *Fig. 3. I guess that rogue waves with heights below some level were not processed. May it be the reason why the maxima of the histograms for $H_{1/3}$ and H_{max} correspond to the minimum values of $H_{1/3}$ and H_{max} for usual series, but not for rogue wave series?*

What is N? I suspect, the total number of rogue wave samples should be much smaller than the number of ordinary wave samples, but do not see this in the figure.

Reply: Thank you for bringing this up. In Fig. 3, N denoted the number of samples. We have changed the y-axis label to 'Number of samples' to make it clearer. The reviewer is correct that only a subset of non-rogue samples was considered for the comparison. The rogue wave samples were collected from 2006 to 2017, while the non-rogue wave samples were manually selected from the year 2012. We selected the wave samples of non-rogue waves with extreme heights ($H_{max} \geq 5m$) and then added randomly chosen non-rogue samples to make the number of these samples similar to that of the rogue wave samples. The procedure is described in the subsection 'Data analysis of wave samples with four types of nonlinear spectra: stable, small and large breather, soliton'.

9. *Line 209. The Hilbert transform is known to be an inaccurate method for constructing envelopes of nonlinear Stokes waves due to the phase-locked nonlinear harmonics. Please comment on that.*

Reply: Thank you for this interesting question. Assuming a narrow-band wave train, the Hilbert transform is accurate to same order as the NLSE itself [2, Eq. 38]. From this point of view, it seems to be adequate for our analysis, and indeed it is commonly used when the NLSE-NFT is applied in the literature. As you point out, improved formulas for the reconstruction of the imaginary part of the envelope have been proposed [3, Ch. 3]. These formulas are accurate to higher orders than the NLSE. The paper [3] compares different methods for the reconstruction of the imaginary part with one another, but unfortunately is the plain Hilbert transform not among the evaluated methods. It is therefore difficult to estimate the difference it would make. Furthermore, since our data has been measured in the field, the buoy tends to underestimate wave heights of large waves resulting in data uncertainty [4]. The uncertainty may change the results more than higher order corrections to the Hilbert transform method. Thus, plain reconstruction tests as in [3] may not tell the whole story. Just judging from the results in [3], it appears that the reconstructed surface elevations differ from the measured wave height in particular of rogue waves, but for the aforementioned reasons it is difficult to say how much this affects our results.

That being said, we would like to point out that wave and crest heights in our paper were directly determined from the measured surface elevations whenever possible. There are only two aspects where our main results rely on the envelope: the classification of the samples into the four types (stable modes, small breathers, large breathers and soliton), and the determination of the amplitude of the dominant nonlinear mode.

Regarding the classification: The dominant mode is by definition the strongest one. A second order correction is therefore not likely to change its type. We thus do not expect that the classification of the samples into the four types would change much if the Hilbert transform is replaced with a higher order

method. For these two reasons, in the Figs. 7 to 11 at most a few data points, if any, could change their marker (the positions of the data points are directly determined from the surface elevation).

Regarding the amplitudes of the nonlinear modes: Using a different method than the Hilbert transform might change the amplitudes of the nonlinear modes, which are computed w.r.t. the envelope. At the moment, we do not have the numerical means to reconstruct surface elevations for individual nonlinear modes. (It is a non-trivial problem.) The maximal amplitude of the nonlinear mode does not have to correspond to half the wave height, especially for unstable modes (breathers and solitons). This might impact the curves shown in Fig. 13. We have therefore added a plot of the amplitude of the largest nonlinear mode over the crest height (in addition to the wave height) in the new version, but the trends look very similar.

We have also added the following text in the subsection "Data normalization of deep water waves for the nonlinear Fourier transform": "The Hilbert transform method is accurate to the same order as the NLSE for narrowband wave-trains [93, Eq. 38], and thus commonly used for NLFT-NSE analyses [48, 73, 94]. However, we remark that improved methods for the computation of the envelope, in particular for rogue waves, have been proposed [95, 96].".

10. *Sec. "Data normalization of ocean waves in finite depth".*

I believe, the depth of 5 km well corresponds to the deep water condition. It is straightforward to estimate that the dimensionless depth parameters are $k_p h = 200$ *for the most typical period* $T_p = 10$ *s and* $k_p h = 50$ *for the longest period according to Fig. 3,* $T_p = 20$ *s. Therefore there is no need to introduce the finite-depth theory, the water is effectively infinitely deep.*

In Eq. (11) the 'nonlinear parameter' ρ *should contain* $\nu(\nu)$ *not* $\nu(v)$*. The relations are just linear scaling of variables, which reduces Eq. (7) to Eq. (1). Therefore the sentence "By applying the multi-dimensional chain rule and substituting* $A = \rho^{-1}$ *in the temporal NLSE, one obtains the normalized temporal NLSE (1)" is not understood at all.*

To conclude, this section may be of a few lines length, no longer.

Reply: We agree that the infinite-depth coefficients will be very close to the finite-depth versions, but since we used the latter for the numerical computations, we would like to keep them here for reproducibility reasons.

We have corrected the typo in Eq. (11).

After substituting $A = \rho^{-1}u$ (in the first submission, the u was missing in this formula due to a typo) in the temporal NLSE

$$i[C_g^{-1}A_t + A_x] + \mu C_g^{-3}A_{tt} + \nu C_g^{-1}|A|^2 A = 0,$$

one only arrives at

$$i\mu^{-1}C_g^3[C_g^{-1}u_t + u_x] + u_{tt} + 2|u|^2 u = 0,$$

which is not yet the normalized NLSE $iu_X + u_{TT} + 2|u|^2 u$. In order to get there, one uses the multi-dimensional chain-rule:

$$u_x = u_X X_x + u_T T_x = \frac{\mu}{C_g^3} u_X - C_g^{-1} u_t,$$

$$u_t = u_X X_t + u_T T_t = 0 + u_T, \quad u_{tt} = \cdots = u_{TT}.$$

However, we agree that just mentioning this in passing might be more confusing than helping. We therefore no longer refer to the multi-dimensional chain rule as the details can be found in the reference that is given in the text.

11. *The purpose why the parameter ρ is introduced is not clear (it seems to be unused below?). The relations (11) do not produce non-dimensional variables u, X, T, the variables are still dimensional. In particular, the parameter ρ is dimensional, m–1c–1, if the envelope $A(x,t)$ describes the surface displacement, as usually. Therefore, the role of the transformation (11) is not clear.*

Reply: Thank you for the question. This seems to be a question of terminology. We never claimed that the variables in the normalized NLSE are dimensionless. Normalized here only refers to the fact that the physical coefficients no longer appear in the equation. The role of the transformation (11) is only to get into the normalized form in order to be able to apply the NLSE-NFT, which assumes this case. We added a sentence to make this clear.

12. *Sec. "Rogue wave criterion and its formation" is not well written. It is discussed several times within a few lines that the down-crossing method is used, that $H_{1/3}$ is the significant wave height, etc*

Reply: Thanks for your comment. We have revised the section in the new version.

13. *Line 259. Right before, the case of an envelope soliton in Fig. 4b is discussed, which is known to be a stable structure. Then, the sentence "Arbitrarily small perturbations can free the trapped auxiliary spectrum points ... so that the solution is unstable to perturbations" is not clear.*

Reply: Thank you for pointing this out. While the soliton seems to have a double point, it is not the case. It is just a very short spine. We now clarify this in the text. We also added an additional row in Fig. 4 that shows the hyperelliptic modes. For the soliton, one can see that the auxiliary spectrum is not trapped.

14. *Lines 267-276. Honestly, I understood almost nothing. There are many words in comas, what probably should mean some lack of rigor in the description. All together, this looks descriptive and uncertain. The paragraph above (lines 248-266) is a bit better. The eigenvalues λ in Fig. 4 come 'from the sky'.*

I see that the topic is mathematically complicated and cannot be fully explained in a few lines, but in the present form the description does not give any idea of origins of the presented details of the theory (see also some more critical remarks below).

Reply: Thank you for bringing this up. It is indeed complicated material and we appreciate the honest feedback. We have significantly revised the subsection "Interpretation of the nonlinear Fourier spectrum" and hope that it is now easier to digest.

15. *Line 279. I do not understand how the degenerate and non-degenerate points may be distinguished in Fig. 4b.*

Reply: As already mentioned, we now also plot the hyperelliptic modes, so that the reader can now see whether a part of the auxiliary spectrum is trapped or not. We furthermore now also mention a theoretical result: degenerate points can only occur on top of spines or on the real axis. (The degenerate modes on the real axis are not shown in the plots because they are known not to contribute to the signal. They also cannot cause any instability. These results are now also mentioned in the revised text.)

16. *Line 285. The expression for the soliton solution is obviously wrong. Should be "sech(T)?"*

Reply: Thanks for spotting this. We have corrected this mistake in the new version.

17. *Line 287. The description 'The two points are however distinct. Since they are very close, the spine cannot be seen in the Fig. 4d' is unclear. In Fig. 4b the points are $\pm i$ (not very close), though $\pm i/2$ in Fig. 4d are 'too close' ?..*

A related question arises when the cases 3 and 4 of the nonlinear spectrum classification are discussed: a quantitative difference between a 'large amplitude breather' and a 'soliton' is not presented.

Reply: The decision was made on visual inspection. When both endpoints of the spine appeared to be at the same location, the nonlinear mode was considered to be a soliton. We added the following text to the manuscript to clarify this: "An unstable mode was considered a soliton if, based on visual inspection, the spine reduced to a single point in the nonlinear spectrum."

18. *Line 289. The sentence "The well-known breather solutions of the NLSE with periodic boundary conditions include Akhmediev breather, Peregrine solitons, Kuznetsov-Ma solitons" is inaccurate. Either Akhmediev of Kuznetsov breather is periodic along the chosen variable. The Peregrine breather is not periodic at all.*

Reply: Thank you for your comment. You are of course correct. We changed that part to 'The well-known breather solutions of the NLSE include Akhmediev breathers, Peregrine breathers and Kuznetsov-Ma breathers. These solutions stand as exemplary prototypes for the modeling of rogue waves in various nonlinear media.'.

19. *Line 294. What are α_3, ρ, η??? In addition, α_1 andα_2 appears a few lines below, which are not explained at all...*

Reply: Thank you for your comment. We have added the explanation 'A- and B-type breathers are specified by parameters known as α_3, ρ, and η, and α_1, α_2, and α_3, respectively.' in the revised version.

20. *Fig. 4. Please add the legend for the main points and spines similar to as done in Fig. 6.*

Reply: We have added a legend in the new version.

21. *Fig. 6. Coefficients of the NLSE are written in terms of the carrier wavenumber and frequency, while the wave spectrum is assumed to be narrow. The frequency band in Fig. 6 is a few octaves. How is it possible? How k_0 and ω_0 were calculated? They are key parameters of the NLSE which specify whether wave groups are stable or not. The applied method imposes periodic boundary conditions, though they are not. How do you make the data periodic?*

Reply: Thank you for your questions. Following the book of Osborne [5, Ch. 13.3], the peak frequency from the linear Fourier spectrum was used as carrier frequency ω_0. k_0 was then calculated from the dispersion relation $\omega_0^2 = gk_0$.

As discussed in the introduction do real ocean waves usually violate both assumptions of the NLSE, narrow-bandedness and uni-directionality. This is also the case for our data. Therefore, as discussed in the manuscript, the identified nonlinear modes cannot be expected to be long-lived.

The effect of deviations from uni-directionality on the NLSE-NFT are thereby much less understood at the moment than deviations from narrow-bandedness. This is why we assess the effect of the directional spreading by repeating our analyses for the parts of the data with the lowest directional spreading.

The effect of applying the NLSE-NFT to broadband signals is, in contrast, quite clear. Some authors remove the low-frequency part with a linear filter before performing the NLSE-NFT [6], but in our experience removing low-frequency parts has negligible effects on the dominant nonlinear Fourier mode that we are interesting in in this paper (which is always close to the peak frequency in our data). In fact, we recently demonstrated this effect explicitly for soliton modes in the paper [7]. We therefore did not apply linear high-pass filters to our data.

The issue that measured ocean waves are rarely periodic of course also arises with conventional linear methods based on the fast Fourier transform (FFT) algorithm. The common way to deal with this issue is windowing, where a so-called window function such as the Hamming window is multiplied with the time series before the FFT is computed. Many windows dampen the edges of the signal so that the periodic extension of the measurement is continuous. The effect of windowing on the FFT is very well-understood, it results in a convolution with the Fourier transform of the window in the frequency domain, which smears out the spectrum. In this work, we considered a rectangular window, which from a practical point view means that the measurement is left unchanged. For linear analyses, this window is not ideal because it can smear out the spectrum quite a bit. For nonlinear analyses using the NLSE-NFT,

the effect of a rectangular window is however at least somewhat understandable from a mathematical point of view. It has also been investigated numerically for non-periodic signals in the form of a "truncation error". In contrast, the impact of other windows on the NLSE-NFT has never been investigated to the best of our knowledge. We therefore chose to stick to a simple rectangular window.

22. *Fig. 7. Please make the caption more precise: "... based on the four types of nonlinear spectra."*

Reply: We have revised the caption in the new version.

23. *Line 403. Based on the preceding discussion, I assume that the sentence "This suggests that the existence of unstable modes in nonlinear spectra is related to large waves in real ocean..." is not fully representative. It is correct for a given Tp, though the authors demonstrate a strong dependence on T_p as well. I believe that the wave steepness $\epsilon \propto kH \propto H/T^2$ may be an appropriate governing parameter for the cases of 'solitons' and 'large amplitude breathers' in Fig. 7a,b. It gives a square-root dependence $T \sim H_{1/3}$ which seems to correspond to the observational data (see 'solitons' in Fig. 7a,b).*

This remark also influences the statements in lines 507-508 and 539-541.

Reply: Thank you for this suggestion. The relationship between the maximum wave height $H_{\max}$ and the square of the peak period T_p^2 is shown in the figure below. In agreement with the reviewer's comments, the results clearly demonstrate that the relation between $H_{\max}$ and T_p^2 is approximately linear for small breathers, large breathers, and solitons, with a slope that depends on the type. We have included the plot and an explanation in the revised version and extended the statements marked by the reviewer.

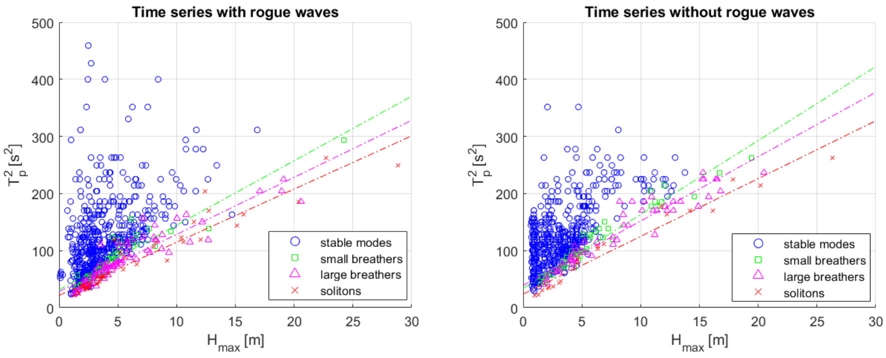

Figure 1: Relationship of the four types of nonlinear spectra to wave steepness (a) Rogue wave cases (b) Non-rogue wave cases.

24. *Line 419. The text "within our the limits of our data set" is obviously corrupted.*

Reply: Thank you for the comment. We have removed this formulation in the new version.

25. *Line 420. Please, give explicitly the range of directional spreads and the method how the angular spectrum width was calculated.*

Reply: We have added an explanation of how the directional spreading is calculated in the revised version. The range of the directional spreads can be found in Fig 3 based on the eqution (13).

26. *Line 425. What does "the largest unstable modes" mean? Is it about the class 4?*

Reply: Thanks for this comment. We have revised the sentence to "In this section, we investigate whether the unstable mode (i.e., small breather, large breather or soliton) with the largest amplitude in the nonlinear spectrum can be attributed to rogue waves in time series.". Please check the revised manuscript.

27. *Line 429. Here and in other examples the information about $H_{1/3}$ and H_{max} is given but not about T_p. Please indicate T_p.*

Reply: We have added the T_p information in the revised version.

28. *Line 431. It is not clear from the description that all the frames are centered at the extreme wave event at $t \approx 218s$. The frames in Fig.9a may be seen only from a monitor when the page is magnified. Please improve the representation.*

Reply: Thanks for spotting this. We have added the sentence 'The window is initially positioned at the center of the rogue wave. If the window extends beyond the bounds of the time series, it is subsequently adjusted by moving it left and right within the time series.' in the new version.

29. *Line 475. The sentence "The finding that for soliton type spectra, the soliton is found in the rogue wave has been confirmed in all but one cases" is repeated twice.*

Reply: Thank you. We have fixed this in the new version.

30. *Line 484. The sentence "We therefore relate the amplitude of the largest nonlinear mode to the maximum wave height of the time series with rogue waves in a scatter plot in Fig. 10" is not accurate: maximum crest height is plotted in Fig. 10, not the maximum height.*

In fact, there is more sense to plot amplitudes of the maximum nonlinear modes in Fig. 10 versus maximum height (not crest height). The wave crest height of nonlinear Stokes waves may be significantly larger than the half-height of the wave due to the phase-locked bound waves. This effect is not taken into account by the authors when the complex envelope is obtained from the surface displacement. The second-order bound waves are effectively eliminated in the wave height quantity.

Reply: Thank you for the comment. We now provide two figures of both wave

height and crest height in the revised version. Both figures show similar trends.

31. *Line 493. Please paraphrase "higher crest heights".*

Reply: Thank you. We have revised this in the new version.

References

[1] Teutsch, I., Brühl, M., Weisse, R. & Wahls, S. Contribution of solitons to enhanced rogue wave occurrence in shallow depths: a case study in the southern north sea. *Natural Hazards and Earth System Sciences* **23**, 2053–2073 (2023).

[2] Slunyaev, A. A high-order nonlinear envelope equation for gravity waves in finite-depth water. *Journal of Experimental and Theoretical Physics* **101**, 926–941 (2005).

[3] Slunyaev, A., Pelinovsky, E. & Guedes Soares, C. Reconstruction of extreme events through numerical simulations. *Journal of Offshore Mechanics and Arctic Engineering* **136**, 011302 (2014).

[4] Benetazzo, A. *et al.* Observation of extreme sea waves in a space–time ensemble. *Journal of Physical Oceanography* **45**, 2261–2275 (2015).

[5] Osborne, A. *Nonlinear Ocean Waves and the Inverse Scattering Transform* (Academic Press, 2010).

[6] Osborne, A. R., Resio, D. T., Costa, A., Ponce de León, S. & Chirivì, E. Highly nonlinear wind waves in currituck sound: dense breather turbulence in random ocean waves. *Ocean Dynamics* **69**, 187–219 (2019).

[7] de Koster, P. & Wahls, S. Fast and reliable detection of significant solitons in signals with large time-bandwidth products. *Journal of Lightwave Technology* (2023).
