# Supplementary material for: Nonlinear Fourier classification of 663 rogue waves measured in the Philippine Sea
Source: PLoS One. 2024 May 14;19(5):e0301709. doi: 10.1371/journal.pone.0301709 (PMC11093313; doi:10.1371/journal.pone.0301709)
Supplement: Attachment — Submitted filename: Report 2.pdf [file pone.0301709.s004.pdf]

Review on the revised manuscript PONE-D-23-29589_R1

"Nonlinear Fourier Classification of 663 Rogue Waves Measured in the Philippine Sea"
by Y.-C. Lee, M. Bruhl, D.-J. Doong, S. Wahls
submitted for publication in PLOS ONE

I find the revised version of the text greatly improved. However, few more points should be clarified / fixed before the paper may be recommended for publication.

1. Line 6 of the abstract: repetition "In addition <…> can additionally".
2. Line 156: please check if the sentence "The results presented here were…" is correct.
3. Line 203: It is said that the depth is 5000 m, though it was indicated 5500 m in line 181. Please make consistent.
4. Line 280: repetition "contain these cases as special cases".
5. Line 313: references to panes (k) and (l) are wrong; should be (h) and (i). Please check.
6. Line 335: references to panes (d), (f) and (h) are wrong; should be (e), (h) and (k). Please check.
7. According to the description in the text, the *main spectrum* is represented by complex numbers E_k (Eq. (2)) plotted in Fig. 4 with blue circles. No explanation is given how the *spines* appear in Fig. 4.
8. Line 370. Frankly speaking, I cannot understand how the statement "*The difference between the spectral components of sine waves and Stokes waves is that the main spectrum points of sine waves are connected by vertical spines, and the main spectrum points of Stokes waves are connected by distorted spines*" may be true. As I may understand, in the present context the difference between sinusoidal waves and Stokes waves is in the presence of phase-locked (bound wave) components which modify the wave shape. The NLS equation describes only the spectral band of the carrier, thus does not contain information about bound waves. Therefore, the distorted spines should reflect something different. I guess, distorted spines may show a significant nonlinear correction to the frequency (?), what is an implicit sign of a Stokes wave.
9. Figure 9 is first mentioned before Fig. 8 (lines 465 and 470 respectively).
10. Line 626. The conclusion "*Our results therefore do not suggest a connection between the BFI's and the rogue wave probability*" is probably not accurate. Such a statement could be drawn based on the scatter *AI* vs *BFI*, which is not present in the paper. Based on Fig. 11, one may say that the maximum wave height grows when *BFI* increases, thus this index may be useful.
