# Supplementary material for: Nonlinear Fourier classification of 663 rogue waves measured in the Philippine Sea
Source: PLoS One. 2024 May 14;19(5):e0301709. doi: 10.1371/journal.pone.0301709 (PMC11093313; doi:10.1371/journal.pone.0301709)
Supplement: Attachment — Submitted filename: PLOS_ONE_authors_response_v2.pdf [file pone.0301709.s005.pdf]

Authors Response to the Reviewer Comments

Y.-C. Lee, M. Brühl, D.J. Doong and S. Wahls

Dear Professor Malomed,

Thank you again for helping us on the submission of our manuscript "Nonlinear Fourier Classification of 663 Rogue Waves Measured in the Philippine Sea" to PLOS ONE. We appreciate the time and effort that you and the reviewer have dedicated to providing feedback on our manuscript. Please find our point-by-point response to the reviewers' comments and concerns below.

Reply to Reviewer

I find the revised version of the text greatly improved. However, few more points should be clarified / fixed before the paper may be recommended for publication.

Reply: Thank you for your positive feedback on our revised manuscript. We appreciate your feedback and will promptly address the following comments:

1. *Line 6 of the abstract: repetition "In addition ... can additionally".*

Reply: Thanks for the comment. We have revised the sentence to

> "Next to the usual linear Fourier modes, NFTs can additionally uncover nonlinear Fourier modes in time series that are usually hidden."

2. *Line 156: please check if the sentence "The results presented here were..." is correct*

Reply: Thanks for pointing this out. We have improved the grammar in this sentence. It now reads

> "The results presented in this paper were obtained using version 0.4.1 of the library [1]. More details on FNFT are available online at https://github.com/FastNFT/FNFT."

3. *Line 203: It is said that the depth is 5000 m, though it was indicated 5500 m in line 181. Please make consistent.*

Reply: Thank you for the comment. The water depth there is actually around 5000 m. We have corrected the text.

4. *Line 280: repetition "contain these cases as special cases".*

Reply: We have revised it to

> "Recently, large families of breathers that contain these solutions as special cases were investigated in the Refs. [105,106]".

5. *Line 313: references to panes (k) and (l) are wrong; should be (h) and (i). Please check.*

Reply: Thank you for noticing these typos. We have fixed them.

6. *Line 335: references to panes (d), (f) and (h) are wrong; should be (e), (h) and (k). Please check.*

Reply: Thanks for your comment. We have fixed this mistake.

7. *According to the description in the text, the main spectrum is represented by complex numbers Ek (Eq. (2)) plotted in Fig. 4 with blue circles. No explanation is given how the spines appear in Fig. 4.*

Reply: Thank you for bringing this up. To clarify the origin and role of the spines, we extended the final paragraph of the subsection "Operation of periodic NLSE-NFT" as follows.

> "The main spectrum consists of the E_k, which are connected by curves known as spines. Just like the main spectrum, the spines remain constant during propagation with respect to the NLSE. In principle, spines are redundant because the signal is already uniquely specified by the main and auxiliary spectrum together with the sheet indices. However, they play an important role when the Riemann theta form of the solution in (6) is computed. Spines provide valuable information about the general characteristics of the solution $u(X, T)$, as they impose topological constraints on the trajectories of the auxiliary spectra $\mu_k(X, T)$ [2, p. 49]. Like the main and auxiliary spectrum, the spines can be found by spectral analysis of the Zakharov-Shabat operator."

8. *Line 370. Frankly speaking, I cannot understand how the statement "The difference between the spectral components of sine waves and Stokes waves is that the main spectrum points of sine waves are connected by vertical spines, and the main spectrum points of Stokes waves are connected by distorted spines" may be true. As I may understand, in the present context the difference between sinusoidal waves and Stokes waves is in the presence of phase-locked (bound wave) components which modify the wave shape. The NLS equation describes only the spectral band of the carrier, thus does not contain information about*

bound waves. Therefore, the distorted spines should reflect something different. I guess, distorted spines may show a significant nonlinear correction to the frequency (?), what is an implicit sign of a Stokes wave.

Reply: Thank you for these comments. The Stokes waves turn into sine waves as their amplitudes become smaller and thus, indeed, nonlinear effects decrease. At the same time, the spines straighten. We are not aware of a mathematical derivation of this phenomenon, but the effect has been consistently observed in the literature. We have added the following clarification to the paper.

> "(The Stokes waves turn into sine waves for small amplitudes. The spines become vertical lines in that case. See e.g. [3, Fig. 13] or compare Figs. 3 and 4 in [1].)"

9. *Figure 9 is first mentioned before Fig. 8 (lines 465 and 470 respectively).*

Reply: Thank you for noticing this. We have changed the order of two paragraphs.

10. *Line 626. The conclusion "Our results therefore do not suggest a connection between the BFI's and the rogue wave probability" is probably not accurate. Such a statement could be drawn based on the scatter AI vs BFI, which is not present in the paper. Based on Fig. 11, one may say that the maximum wave height grows when BFI increases, thus this index may be useful.*

Reply: Thank you. To make the argument clearer, we have revised that part to

> "If the BFIs were correlated with the rogue wave probability, one would expect higher BFIs for the rogue wave data. Since that does not appear to be the case here, our results do *not* suggest a connection between the BFIs and the rogue wave probability."

References

[1] Wahls, S., Chimmalgi, S., Prins, P. J. & Brehler, M. Fastnft/fnft: Version 0.4.1 (2020). URL `https://doi.org/10.5281/zenodo.3941976`.

[2] Forest, M. G. & Lee, J.-E. Geometry and modulation theory for the periodic nonlinear schrodinger equation. In *Oscillation Theory, Computation, and Methods of Compensated Compactness*, 35–69 (Springer, 1986).

[3] Osborne, A. R., Resio, D. T., Costa, A., Ponce de León, S. & Chirivì, E. Highly nonlinear wind waves in currituck sound: dense breather turbulence in random ocean waves. *Ocean Dynamics* **69**, 187–219 (2019).
